# Anxiety increased among children and adolescents during pandemic-related school closures in Europe: a systematic review and meta-analysis

**DOI:** 10.1186/s13034-023-00612-z

**Published:** 2023-06-21

**Authors:** Helena Ludwig-Walz, Indra Dannheim, Lisa M. Pfadenhauer, Jörg M. Fegert, Martin Bujard

**Affiliations:** 1 Federal Institute for Population Research (BiB), Wiesbaden, Germany; 2 Regional Innovative Centre of Health and Quality of Live Fulda (RIGL), Fulda University of Applied Sciences, Fulda, Germany; 3 Department of Nutritional, Food and Consumer Sciences, Fulda University of Applied Sciences, Fulda, Germany; 4 Chair of Public Health and Health Services Research, IBE, Faculty of Medicine, LMU Munich, Munich, Germany; 5 Pettenkofer School of Public Health, Munich, Germany; 6 Department for Child and Adolescent Psychiatry and Psychotherapy, University Medical Center, Competence Domain Mental Health Prevention, Ulm, Germany; 7 Institute of Medical Psychology, Medical Faculty, University Heidelberg, Heidelberg, Germany

**Keywords:** Anxiety, COVID-19, Child, Adolescent, Europe, Meta-analysis, Communicable disease control, Lockdown, Evidence-informed decision-making, Pandemic preparedness, Health policy

## Abstract

**Background:**

Considering the heterogenous evidence, a systematic review of the change in anxiety in European children and adolescents associated with the COVID-19 pandemic is lacking. We therefore assessed the change compared with pre-pandemic baselines stratified by gender and age as well as evaluated the impact of country-specific restriction policies.

**Methods:**

A registration on the ‘International Prospective Register of Systematic Reviews’ (PROSPERO) occurred and an a priori protocol was published. We searched six databases (PubMed, Embase, PsycINFO, Cochrane Central Register of Controlled Trials, Web of Science, WHO COVID-19) using a peer-reviewed search string with citation tracking and grey literature screening. Primary outcomes were: (1) general anxiety symptoms; and (2) clinically relevant anxiety rates. We used the Oxford COVID-19 Stringency Index as an indicator of pandemic-related restrictions. Screening of title/abstract and full text as well as assessing risk of bias (using the ‘Risk of Bias in Non-randomized Studies of Exposure’ [ROBINS-E]) and certainty of evidence (using the ‘Grading of Recommendations Assessment, Development and Evaluation’ [GRADE]) was done in duplicate. We pooled data using a random effects model. Reporting is in accordance with the Preferred Reporting Items for Systematic review and Meta-Analysis (PRISMA) statement.

**Results:**

Of 7,422 non-duplicate records, 18 studies with data from 752,532 pre-pandemic and 763,582 pandemic participants met full inclusion criteria. For general anxiety symptoms the total change effect estimate yielded a standardised mean difference (SMD) of 0.34 (95% confidence interval [CI], 0.17–0.51) and for clinically relevant anxiety rates we observed an odds ratio of 1.08 (95%-CI, 0.98–1.19). Increase in general anxiety symptoms was highest in the 11–15 years age group. Effect estimates were higher when pandemic-related restrictions were more stringent (Oxford Stringency Index > 60: SMD, 0.52 [95%-CI, 0.30–0.73]) and when school closures (School Closure Index ≥ 2: SMD, 0.44 [95%-CI, 0.23–0.65]) occurred.

**Conclusion:**

General anxiety symptoms among children and adolescents in Europe increased in a pre/during comparison of the COVID-19 pandemic; particularly for males aged 11–15 years. In periods of stringent pandemic-related restrictions and/or school closures a considerable increase in general anxiety symptoms could be documented.

PROSPERO registration: CRD42022303714.

**Supplementary Information:**

The online version contains supplementary material available at 10.1186/s13034-023-00612-z.

## Background

Mental disorders are important causes of disease burden among children and adolescents [1, 2]. Even before the COVID-19 pandemic, the burden of disease study highlighted that anxiety disorders were the most prevalent condition in 2019 among young people in Europe. Among mental health conditions, such disorders represented a leading cause of years lived with disability [2, 3]. In this regard, a link can be drawn between the non-treatment or undertreatment of anxiety disorders in childhood and adolescence and mental illnesses in adulthood, such as anxiety, depression and substance use disorders [4, 5]. Anxiety is generally defined as feelings of concern that appear to have no obvious cause, but are sufficiently persistent and severe to affect daily life [6]. With the onset of the COVID-19 pandemic, the implementation of a broad range of public health and social measures (PHSM) [7] served to exacerbate many determinants of poor mental health. In particular, the environment of children and adolescents has been changed considerably by PHSM, which comprise school and leisure facilities closing, fewer peer interactions, changes in the family system as a result of the requirement to work from home, and quarantine orders [7–9]. As already known from previous studies [10–13], such changes can lead to serious impairments in young people’s mental health. To date, the impact of the COVID-19 pandemic on anxiety has been assessed primarily for the adult population [14–16] or its global prevalence for children and adolescents [17–19]. Existing European studies with a pre-pandemic baseline showed heterogeneous results [20–23]. However, a deeper understanding of changes in anxiety symptoms in the young population group is lacking, especially for the European continent.

An up-to-date examination of changes in anxiety symptoms among children and adolescents is therefore imperative and of great public health (PH) relevance in order to counteract suboptimal developments [2]. An analysis of the changes in the European continent means that the lack of an evidence base in the subgroup-stratified summary among children and adolescents can be rectified. It also allows for the use of a quasi-experimental design by analysing the impact of heterogeneous pandemic-related interventions in the European countries. Hence, the aim of this systematic review and meta-analysis is to identify, critically assess, summarise, and determine the certainty of evidence (CoE) regarding the impact of the COVID-19 pandemic on anxiety among children and adolescents in Europe compared with the pre-pandemic baseline. Thereby, it aims to provide information about the relevance of pandemic-related restrictions which will contribute to the analysis and the lessons learned from the immediate restrictions taken to safeguard the population in various European countries.

## Methods

This systematic review and meta-analysis is reported according to the Preferred Reporting Items for Systematic Reviews and Meta-analyses (PRISMA) [24] statement (Additional file 1: Table S1). Our protocol is registered on the ‘International Prospective Register of Systematic Reviews’ (PROSPERO; CRD42022303714) [25] and was published a priori [26]; any deviations from the original review protocol are presented in Additional file 1: Table S2.

### Data sources, search strategy and eligibility criteria

We searched for published articles in six electronic databases (PubMed, Embase, PsycINFO, Cochrane Central Register of Controlled Trials, Web of Science, WHO COVID-19 database [including pre-prints]), up to 18 March, 2022. Additionally, we enlarged our searches by examining previous systematic reviews and meta-analysis on the same topic, checking reference lists in included studies and searching relevant grey literature sources such as reports issued by key organisations and abstracts of relevant conferences up to 16 April, 2022; more information on the screened key organisations and conferences is provided in Additional file 1: Table S3.

We developed the search strategy according to the Population–Exposure–Comparison–Outcome (PECO) [27] scheme and included the following key search terms: children and adolescents (population), COVID-19 (exposure) and anxiety (outcome). The availability of a pre-pandemic baseline (comparison) was assessed manually. The six tailored search strategies can be found in Additional file 1: Table S4. The search strategy was reviewed by a search specialist using the evidence-based checklist ‘Peer Review of Electronic Search Strategies’ (PRESS) [28].

Our pre-defined eligibility criteria were equally defined according to the PECO [27] scheme:

- Population: Children and adolescents ≤ 19 years, living in the WHO European region [29].
- Exposure: Participation in survey during the COVID-19 pandemic.
- Comparison: Pre-pandemic baseline.
- Primary outcomes: Measurements of general anxiety symptoms or clinically relevant anxiety rates; no secondary outcomes were considered.

We excluded studies undertaken in children and adolescents with pre-existing psychiatric diagnoses. No limits regarding language and effect measurement were applied, however our search strategy was designed and run in English. Publications drawing upon the same study population and measurement time points were included as one item. When measurement time points varied during the COVID-19 pandemic, each measurement time point was considered individually.

### Selection process and data extraction

After deduplication, two reviewers (HLW, ID) used the recommended EPPI reviewer software [30] to independently screen first titles and abstracts, and second full texts, in accordance with the above eligibility criteria. Disagreements or uncertainty about eligibility were resolved through discussion. Reasons for exclusion after full text screening were recorded and are reported in a separate table (Additional file 1: Table S5).

Further, two reviewers (HLW, ID) used piloted extraction forms to independently extract data from one third of the published studies and unpublished data requested from study authors. Remaining data extraction was completed by one reviewer (HLW) and verified by the other (ID). Differences in data extraction were discussed and resolved between the two reviewers. Our data extraction forms, in accordance with a former systematic review [31], included the following items: study information (first author, year of publication, country, study type), population and setting (sample size, % female, age of CA), COVID-19 determinants (time point of data measurement), pre-pandemic baseline (time point of data measurement, link between pre-pandemic population and the population during the pandemic) and outcomes (type of outcome, diagnostic instrument, psychometric properties of the diagnostic instrument, symptom reporter). We defined general anxiety symptoms and clinically relevant anxiety rates as primary outcomes. General self-reported measurements of anxiety were summarised as general anxiety symptoms. Since the measurement instruments and scales used varied considerably, the measurement data was standardised to standardised mean difference (SMD) with a 95% confidence interval (CI); this standardisation is also recommended by the Cochrane Handbook [32]. Measurements with a clinical cut-off or with a clinical diagnostic (International Statistical Classification of Diseases and Related Health Problems [ICD]) were summarised as clinically relevant anxiety rates and reported as odds ratio (OR) with a 95% CI. To describe PHSM restrictions in the measurement time frame of the studies and make them comparable, we used the Oxford COVID-19 Stringency Index [8] and the School Closure Index [8] as indicators. The Oxford COVID-19 Stringency Index consists of nine metrics including school closures, workplace closures and stay-at-home requirements. The index ranges from 0 (no restrictions) to 100 (most stringent restrictions) and was validated [8]. In accordance with the COVIDSurg Collaborative [33], we defined three categories: light restrictions (index < 20), moderate lockdowns (index 20–60) and full lockdowns (index > 60). The School Closure Index represents the handling of school closures and is an incorporated measurement in the Oxford COVID-19 Stringency Index, which was considered separately in our analyses. The index ranges from 0 to 3: 0 describes no restrictions; 1 contains recommended closure or all schools open with alterations resulting in significant differences compared with non-COVID-19 operations; 2 involves closure (only some levels or categories, e.g. just high school, or just public schools); and 3 requires closures at all levels [8]. We defined the cut-offs as ‘no or few alterations compared with a pre-COVID-19 situation’ (index < 2) and ‘partial or full school closure’ (index ≥ 2) [31]. We contacted nearly all study authors and asked to provide further unpublished data on age or gender-stratified data.

### Risk of Bias assessment

Three reviewers (HLW, LMP, ID) independently assessed the risk of bias (RoB) in teams of two using the ‘Risk of Bias in Non-randomized Studies of Exposure’ (ROBINS-E) instrument [34]. For each study, the seven bias domains and a whole RoB assessment was revealed as either low, some concerns, high RoB, or very high RoB [34].

### Data synthesis and statistical analyses

For the meta-analysis, we pooled effect estimates for general anxiety symptoms and clinically relevant anxiety rates in total and analysed different subgroups: gender (female/male), age (11–15, 16–19 years), Oxford Stringency Index (> 60/ ≤ 60) [8] and School Closure Index (≥ 2/ < 2) [8]. We used, where possible, results from adjusted analysis for pooling. If necessary, dichotomous data were transferred to SMD, using the formula recommended by Chinn [35]. Where multiple pre-pandemic measurements were available, the last measurement was used for calculation purposes. We excluded measurements, with combined anxiety/depression scores, from the meta-analysis. Where parent and self-reported data were presented [36], we gave preference to the self-reported data. Furthermore, within the meta-analysis, we grouped the studies according to their RoB rating; low/some concerns (= low) RoB studies and high RoB/very high RoB (= high) RoB studies were summarised both separately and in total. In particular, the pooled effect of the low RoB studies was taken for further interpretation. We used Review Manager 5.4.1 [37] and R Studio 4.2.1 [38] for data entry, statistical analysis, and graph creation. In all meta-analyses, random-effect models and the inverse-variance method with the ‘DerSimonian and Laird’ approach were used.

We investigated heterogeneity by using visual inspection of the forest plots as well as the Chi^2^ test and I^2^ index [39]. If I^2^ > 50%, substantial heterogeneity was presumed. We conducted sensitivity analyses and meta-regression (if ≥ 10 studies per examined variable) to explain substantial heterogeneity [40]. Publication bias was analysed by visually interpreting funnel plots for signs of asymmetry [41] and statistically by calculating the Egger’s test (if ≥ 10 studies) [42].

### Certainty of evidence

We assessed the overall CoE for each outcome using the ‘Grading of Recommendations Assessment, Development and Evaluation’ (GRADE) system and presented it along with the main findings of the review in a ‘Summary of findings’ table, based on a transparent format with defined applied criteria (Additional file 1: Table S6) and a generated evidence profile (Additional file 1: Table S7) [43]. The GRADE tool covers five categories for downgrading (RoB, imprecision, inconsistency, indirectness, publication bias) and three categories for upgrading (magnitude of effects, dose–response relationships, impact of residual confounding). The CoE could be rated as high, moderate, low or very low.

## Results

Our electronic search identified 7,420 non-duplicate records from database searches and additional two grey literature publications. Of these, 51 studies entered full-text screening. After a comprehensive screening process, detailed in the PRISMA flow diagram (Additional file 1: Figure S1), we included 18 studies with 22 effect measures, comprising 16 peer-reviewed studies [20, 22, 23, 36, 44–55], one report [56], and one pre-print [21]. Reasons for exclusion after full-text screening are described in Additional file 1: Table S5.

### Study characteristics

The characteristics of each of the studies that were included are described in Table 1. The total population sample included data from 752,532 pre-pandemic and 763,582 pandemic participants (broken down into general anxiety symptoms: 11,425 pre-pandemic and 13,387 pandemic participants; clinically relevant anxiety rates: 741,107 pre-pandemic and 750,195 pandemic participants). Studies were carried out in a range of countries: four in Germany [21, 44, 45, 56], four in the United Kingdom [22, 23, 36, 55], three in Italy [47–49], two in Spain [51, 52], two in Switzerland [53, 54], and one in Israel [46], one in the Netherlands [20], and one in Norway [50], respectively. Most of the studies measured general anxiety symptoms in spring/summer 2020 (14 effect measures) [20–23, 36, 45–47, 49–51, 53–55], while two effect measurements were conducted in autumn 2020 [21, 22] and three in winter 2020/spring 2021 [21, 51, 52]. Clinically relevant anxiety rates were analysed in four studies [44, 45, 48, 56]. Of the included studies, 17 [20–23, 36, 44–50, 52–56] reported data for children and adolescents over the age of 11 and 11 studies [20, 36, 44, 45, 47–49, 51, 54–56] for children and adolescents under the age of 11. The measurement time point was rated as ‘full lockdown’ (Oxford Stringency Index > 60) in 14 studies [20, 22, 23, 36, 44, 46–54] and partial or full school closure occured in 11 studies (School Closure Index ≥ 2) [20–23, 36, 46–49, 51, 55]. In addition, 12 studies [20–23, 36, 44, 45, 47, 49, 53, 55, 56] provided further study data (generally unpublished gender-stratified and age-stratified data). The effect estimates of the 18 studies that were included are summarised in Additional file 1: Table S8. The RoB assessment revealed a ‘some concerns’ rating for six studies [20, 21, 44, 46, 53, 56], a ‘high RoB’ rating for eight studies [22, 23, 36, 45, 47, 50, 51, 55] and a ‘very high RoB’ rating for four studies [48, 49, 52, 54]. Detailed rating information is provided in Additional file 1: Figure S2 (traffic-light plot) and Additional file 1: Figure S3 (weighted-bar plot).

**Table 1:** Characteristics of the studies included

Study information		Population		Exposure		Comparison		Outcome		Risk of bias
First author, year	Study type, name of the study	Sample size (% female)	Age of study population	Time point during COVID-19 pandemic	Policy indices [8], Mean (min to max)	Time point of pre-pandemic baseline	Link between pre-pandemic and during pandemic population	Type of outcome	Diagnostic instrument; psychometric properties; symptom reporter	
*Germany*										
Ravens-Sieberer, 2022 [21]	Cohort study, German COPSY study	PP: 994 (47.8) DP1: 1,018 (49.6) DP2: 1,073 (49.2) DP3: 1,173 (48.4)	PP: NI DP1: Mean (years) ± SD, 12.3 ± 3.3 DP2: Mean (years) ± SD, 12.7 ± 3.3 DP3: Mean (years) ± SD, 14.8 ± 2.3 Age range (years): 11 to 19	DP1: 5–6/2020 DP2: 12/2020—1/2021 DP3: 9–10/2021	DP1 Stringency Index: 59.7 (57.4 to 59.7) School Closure Index: 2.0 (2.0 to 2.0) DP2 Stringency Index: 83.1 (82.4 to 85.2) School Closure Index: 3.0 (3.0 to 3.0) DP3 Stringency Index: 49.2 (37.0 to 60.2) School Closure Index: 1.3 (1.0 to 3.0)	2017 (nationwide, longitudinal, representative BELLA study)	both study samples (BELLA and COPSY) are representative samples of German children and adolescents	General anxiety symptoms	Screen for Child Anxiety Related Disorders (SCARED); Psychometric properties [69]; Self-reported	Moderate
Witte, 2022 [56]	Cross-sectional study, medical record data from a health insurance company	PP: 533,701 (48.6) PP: 332,945 (48.6; in-patient treatment) DP1: 545,626 (48.6) DP1: 339,361 (48.6; in-patient treatment) DP2: 343,642 (48.5; in-patient treatment)	Age range (years): 5 to 17	DP1: 2020 DP2: 2021	DP1: Stringency Index: 51.8 (0 to 76.9) School Closure Index: 1.6 (0.0 to 3.0) DP2: Stringency Index: 67.0 (46.3 to 85.2) School Closure Index: 1.9 (1.0 to 3.0)	2019	Cross-sectional population samples	Clinically relevant anxiety symptoms	Paediatric visit/ Hospitalisation rate ICD-10: F40/41; NI for psychometric properties (International Statistical Classification of Diseases and Related Health Problems [ICD]); Paediatric reported	Moderate
Kostev, 2021 [44]	Cross-sectional study, medical record data from the Disease Analyzer database (IQVIA)	PP: 206,528 (39.2; prevalence, incidence) DP: 203,742 (39.7; prevalence, incidence)	PP: Mean (years) ± SD, 6.6 ± 4.9 DP: Mean (years) ± SD, 6.7 ± 5.0 Age range (years): 2 to 17	4/2020 to 12/2020	Stringency Index: 62.2 (49.5 to 82.4) School Closure Index: 1.8 (1.0 to 3.0)	4/2019 to 12/2019	Cross-sectional population samples	Clinically relevant anxiety symptoms	Paediatric visit ICD-10: F41; NI for psychometric properties (International Statistical Classification of Diseases and Related Health Problems [ICD]); Paediatric reported	Moderate
Rau, 2021 [45]	Cohort study	PP: 777 (53.3) PP: 777 (53.3)	Mean (years) ± SD, 12.9 ± 2.0 Age range (years): 9 to 17	6–7/2020	Stringency Index: 59.7 (57.4 to 63.4) School Closure Index: 1.9 (1.0 to 3.0)	PP1: 10–11/2019 PP2: 1–2/2020	Same population	General anxiety symptoms Clinically relevant anxiety symptoms Clinically relevant anxiety symptoms	Revised Child Anxiety and Depression Scale (RCADS) with anxiety subscales; Psychometric properties [70]; Self-reported	Serious
*Israel*										
Shoshani, 2021 [46]	2-point survey	PP: 1,537 (52) DP: 1,537 (52)	Mean (years) ± SD, 14.0 ± 2.0 Age range (years): 11 to 17	4/2020	Stringency Index: 77.3 (75.0 to 84.3) School Closure Index: 2.1 (2.0 to 3.0)	9/2019	Same population	General anxiety symptoms	Brief Symptom Inventory 18 (BSI-18) with the subscale anxiety; NI for psychometric properties; Self-reported	Moderate
*Italy*										
Frigerio, 2022 [47]	Longitudinal study, Effect of Depression on Infants (EDI)	PP1: 94 (46.8) PP2: 88 (46.6) DP: 59 (45.8)	PP1: Mean (months) ± SD, 13.7 ± 1.63 PP2: Mean (years) ± SD, 3.5 ± 0.3 DP: Mean (years) ± SD, 4.2 ± 0.6	4–6/2020	Stringency Index: 77.4 (67.6 to 93.5) School Closure Index: 3.0 (3.0 to 3.0)	PP measures 1 and 2 (no detailed information)	Same population	General symptoms, subscale anxious/ depressed	Child Behavior Checklist (CBCL 1½-5), subscale anxious/ depressed; Psychometric properties [71]; Parent-reported	Serious
Davico, 2021 [48]	Cross-sectional study	Psychiatric Emergency Department (ED) visits PP1: 101, (61.4) PP2: 93 (65.6) PP3: 131 (55.0) DP: 50 (54.0)	Median (years) (IQR) PP1: 15.1 (13.2 to 16.6) PP2: 14.1 (11.7 to 16.3) PP3: 14.7 (11.9 to 16.5) DP: 15.7 (13.2 to 16.7) Age range (years): 0 to 18	2–4/2020	Stringency Index: 81.8 (64.4 to 93.5) School Closure Index: 3.0 (3.0 to 3.0)	PP1: 1–2/2019 PP2: 2–4/2019 PP3: 1–2/2020	Cross-sectional population samples	General anxiety symptoms	Hospitalisation rate ED visits due to anxiety; NI for psychometric properties; Paediatric reported	Critical
Crescen-tini, 2020 [49]	Online survey	PP: 721 (48.4) DP: 721 (48.4)	Mean (years) ± SD, 10.1 ± 2.5 Age range (years): 6 to 18	4–5/2020	Stringency Index: 90.2 (75.0 to 93.5) School Closure Index: 3.0 (3.0 to 3.0)	retrospective (final months of 2019)	Same population	General anxiety symptoms	Child Behavior Checklist (CBCL 6–18), subscale anxiety; Psychometric properties [72]; Parent-reported	Critical
*Netherlands*										
Luijten, 2021 [20]	Cross-sectional study	PP: 1,318 (50.1) DP: 813 (54.6)	PP: Mean (years) ± SD, 13.1 ± 3.1 DP: Mean (years) ± SD, 13.4 ± 2.8 Age range (years): 8 to 18	4–5/2020	Stringency Index: 78.7 (78.7 to 78.7) School Closure Index: 3.0 (3.0 to 3.0)	12/2017–7/2018 (2 studies)	PP: 2 representative studies DP: 1 representative study (not same population)	General anxiety symptoms	Patient-Reported Outcome Measurement Information System (PROMIS), CAT V2.0-Anxiety; Psychometric properties [73]; Self-reported	Moderate
*Norway*										
Hafstad, 2021 [50]	Representative longitudinal survey	PP: 3,572 (50.1) DP: 3,572 (50.1)	Mean (years) ± SD, 14.7 ± 4.1 Age range (years): 12 to 16	6/2020	Stringency Index: 42.5 (40.7 to 58.3) School Closure Index: 1.0 (1.0 to 1.0)	2/2019	Same population	General symptoms, anxiety and depression	Hopkins Symptom Checklist (HSCL-10); Psychometric properties [74, 75]; Self-reported	Serious
*Spain*										
Giménez-Dasí, 2021 [51]	3-point survey	PP:206 (51.9) DP1:66 (62.1) DP2: 205 (53.7)	Mean months ± SD, 102.4 ± 20.2 Age range (years): 6 to 11	DP1: 3–4/2020 DP2: 12/2020–2/2021	DP1: Stringency Index: 68.2 (11.1 to 85.2) School Closure Index: 2.6 (0.0 to 3.0) DP2: Stringency Index: 72.2 (68.5 to 78.7) School Closure Index: 1.3 (1.0 to 3.0)	12/2019 to 2/2020	Same population: PP and DP2 Subgroup of children: DP1	General anxiety symptoms	Anxiety scale ‘System of Evaluation of Children and Adolescents’ (SENA) questionnaire; Psychometric properties [76]; Self-reported (partly with parents’ supervision)	Serious
Carrillo-Diaz, 2022 [52]	2-point-survey	213 (54.5)	Mean (years) ± SD, 14 ± 1.9 Age range (years): 11 to 17	9–12/2020	Stringency Index: 67.6 (58.8 to 78.7) School Closure Index: 1.7 (1.0 to 3.0)	9–12/2019	Same population	General anxiety symptoms	A: State Anxiety Scale (STAI-S); Psychometric properties [77]; Self-reported	Critical
*Switzerland*										
Ertanir, 2021 [53]	Longitudinal study, ‘Overcoming Inequalities with Education’ project	PP: 359 (46.2) DP: 314 (43.0)	Mean (years) ± SD, 12.7 ± 0.7 Age range (years): 11 to 15	8–9/2020	Stringency Index: 43.1 (43.1 to 43.1) School Closure Index: 0.0 (0.0 to 0.0)	9–10/2019	Same population	General anxiety symptoms	Hopkins Symptoms Checklist (HSCL-25), subscale anxiety; NI for psychometric properties; Self-reported	Moderate
Borbás, 2021 [54]	Cohort study	PP: 26 (38.5) PP: 26 (38.5)	Mean (years) ± SD, 10.7 ± 2.5 Age range (years): 7 to 17	5/2020	Stringency Index: 69.4 (69.4 to 69.4) School Closure Index: 0.0 (0.0 to 0.0)	3/2018 to 2/2020	Same population	General symptoms, subscale anxious/ depressed	Child Behavior Checklist (CBCL 6–18), subscale anxious/ depressed; Psychometric properties [78]; NI	Critical
*United Kingdom*										
Knowles, 2022 [23]	Cohort study, REACH (Resilience, Ethnicity, and AdolesCent Mental Health)	PP1: 955 (NI) PP2: 943 (NI) PP3: 836 (53.7) DP: 1,069 (54.5)	Age range (years): 12 to 18	5–8/2020	Stringency Index: 71.1 (31.5 to 79.6) School Closure Index: 2.9 (2.0 to 3.0)	PP1: 2016–17 PP2: 2017–18 PP3: 2018–19	Same population	General anxiety symptoms	Generalised Anxiety Disorder Scale (GAD-7), moderate-to-severe anxiety as GAD-7 score ≥ 10; Psychometric properties [79]; Self-reported	Serious
Widnall, 2022 [22]	Longitudinal 3-wave panel survey	PP: 589 (59.2) DP1: 587 (58.8) DP2: 587 (59.8)	Mean (years): 13.2 Age range (years): 13 to 15	DP1: 5/2020 DP2: 10/2020	DP1: Stringency Index: 74.2 (69.4 to 79.6) School Closure Index: 3.0 (3.0 to 3.0) DP2: Stringency Index: 67.9 (60.2 to 75.0) School Closure Index: 3.0 (3.0 to 3.0)	10/2019	Same population: PP and DP1 DP2: Other population	General anxiety symptoms	Hospital Anxiety & Depression Scale (HADS), subscale anxiety; Psychometric properties [80]; Self-reported	Serious
Wright, 2021 [36]	Cohort study, population‐based birth cohort (Wirral Child Health and Development Study)	Self-rated: PP: 187 (46.5) DP: 163 (45.4) Mother-rated: PP: 226 (45.5) DP: 199 (54.8)	Mean (years) ± SD, 12.0 ± 0.4 Age range (years): 10 to 12	6–8/2020	Stringency Index: 67.9 (64.4 to 73.2) School Closure Index: 2.8 (2.0 to 3.0)	12/2019—3/2020	Same population	General anxiety symptoms	Short Spence Anxiety Scale; Psychometric properties [81]; Mother-reported	Serious
Bignardi, 2020 [55]	Cohort study, Resilience in Education and Development (RED) study	School group: 114 (49.1) Lab group: 54 (63.0)	PP: School group: mean (years) ± SD, 8.7 ± 0.6 Lab group: mean (years) ± SD, 8.5 ± 0.7 DP: School group: mean (years) ± SD, 10.5 ± 0.7 Lab group: mean (years) ± SD, 9.4 ± 0.8 Age range (years): 7 to 11	4–6/2020	Stringency Index: 74.9 (67.6 to 79.6) School Closure Index: 3.0 (3.0 to 3.0)	School group: 6/2018 to 3/2019 Laboratory group: 12/2018 to 9/2019	Same population	General anxiety symptoms	Revised Child Anxiety and Depression Scale (RCADS)-short form with anxiety subscales; Psychometric properties [70]; PP: self/parent-reported, DP: parent-reported	Serious

*DP* during pandemic, *ED* Emergency departments, *M* mean, *NI* no information, *PP* pre-pandemic, *SD* Standard deviation

### Meta-analysis of general anxiety symptoms

**Fig. 1:**
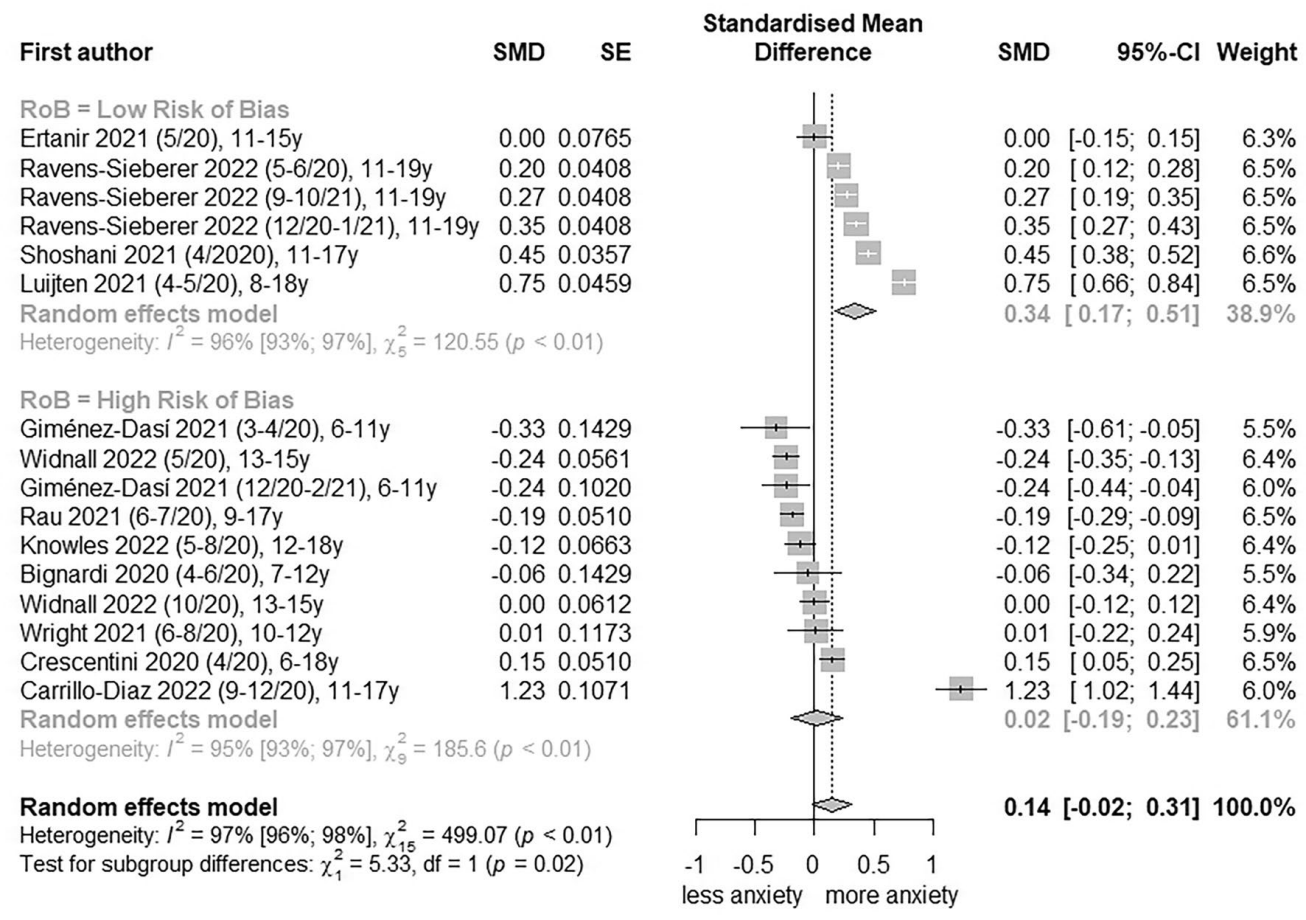
Forest plot of changes in youth general anxiety symptoms comparing before and during COVID-19 pandemic. SE, standard error; SMD, standardized mean differences; RoB, risk of bias; 95%-CI, 95%-confidence interval

For general anxiety symptoms, 12 studies [20–23, 36, 45, 46, 48, 49, 52, 53, 55] were pooled and CoE was graded as ‘very low’ (Table 2; further information in Additional file 1: Table S7). In a pooling of four low RoB studies with six measures, a total change of a SMD of 0.34 (95% CI, 0.17 to 0.51, I^2^ = 96%; Fig. 1) was calculated. Following gender stratification, a SMD of 0.30 (95% CI, 0.12 to 0.49, I^2^ = 90%; Additional file 1: Figure S4) for females and 0.34 (95% CI, 0.07 to 0.60, I^2^ = 95%; Additional file 1: Figure S5) for males in low RoB studies was revealed. Age-stratified pooling was possible for the 11–15 years age category with three studies [20, 21, 53] and five effect measures, and for the 16–19 years age category with two studies [20, 21] and four effect measures. For the 11–15 years age category, the total change effect estimate yielded a SMD of 0.39 (95% CI, 0.18 to 0.60, I^2^ = 93%; Additional file 1: Figure S6). Change effect estimates were also evident for females (SMD, 0.34; 95% CI, 0.19 to 0.49; I^2^ = 71%; Additional file 1: Figure S7) and males (SMD, 0.45; 95% CI, 0.15 to 0.74; I^2^ = 93%; Additional file 1: Figure S8). Pooling within the 16–19 years age category revealed a SMD of 0.24 (95% CI, -0.01 to 0.49, I^2^ = 92%; Additional file 1: Figure S9) in total, a SMD of 0.18 (95% CI, -0.01 to 0.37; I^2^ = 75%; Additional file 1: Figure S10) for females and a SMD of 0.31 (95% CI, -0.02 to 0.63; I^2^ = 92%; Additional file 1: Figure S11) for males.

**Table 2:** Summary of findings

Outcome	Number of studies	Standardised mean difference, 95% CI	Odds Ratio, 95% CI	Summary of findings	Certainty of evidence (GRADE)
General anxiety symptoms	12 studies [20–23, 36, 45, 46, 49, 51–53, 55]	Low risk of bias studies:0.34, 0.17 to 0.51 All studies: 0.14, -0.02 to 0.31		Low risk of bias studies predicted an increase in general anxiety symptoms in the total population, female and male children and adolescents with a dose response-relationship.	⊕ ⊝ ⊝ ⊝ Very low ^a,b,c^
Clinically relevant anxiety rates	4 studies [44, 45, 48, 56]		Low risk of bias: 1.08, 0.98 to 1.19 All studies: 0.99, 0.85 to 1.15	Low risk of bias studies predicted no increase in clinically relevant anxiety rates in the total population and male children and adolescents; however, with partly moderate confidence intervals.	⊕ ⊝ ⊝ ⊝ Very low ^a,b,c^

^a^Downgraded -1 for risk of bias due to some concerns about bias as 66% of the included studies were assessed with serious or critical risk of bias

^b^Downgraded -1 for inconsistency due to a significant chi^2^ test and a substantial high I^2^ test (> 50%), further analysis via subgroup analysis, sensitivity analysis and meta-regression analysis were conducted

^c^Downgraded -1 for indirectness due to moderate confidence intervals and overlap of the line of no effect of the 95% CI in total effect estimate, although a broad sample size

**Fig. 2:**
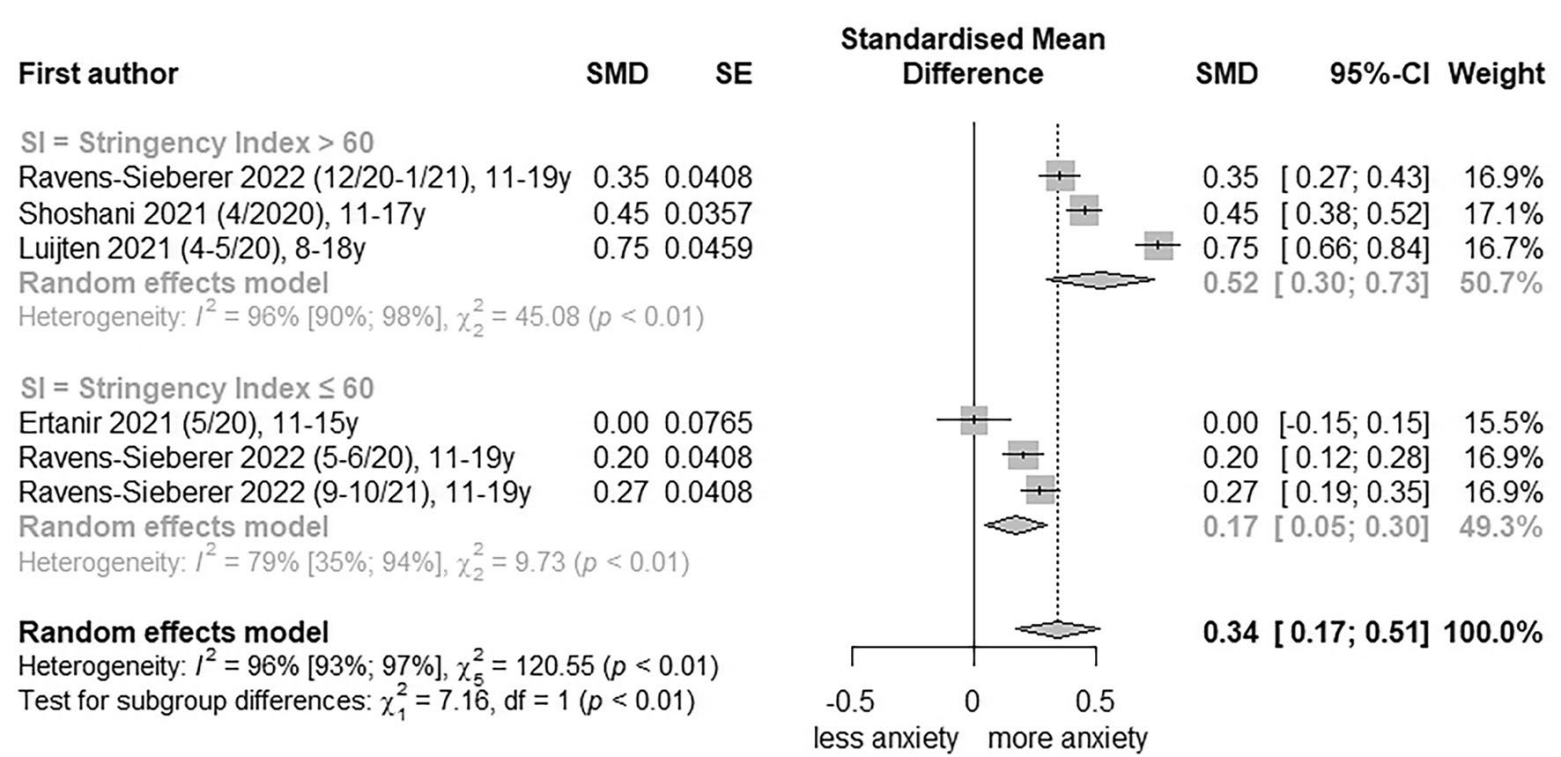
Forest plot of changes in youth general anxiety symptoms comparing Oxford Stringency Index. SE, standard error; SMD, standardized mean differences; SI, stringency index; 95%-CI, 95%-confidence interval

**Fig. 3:**
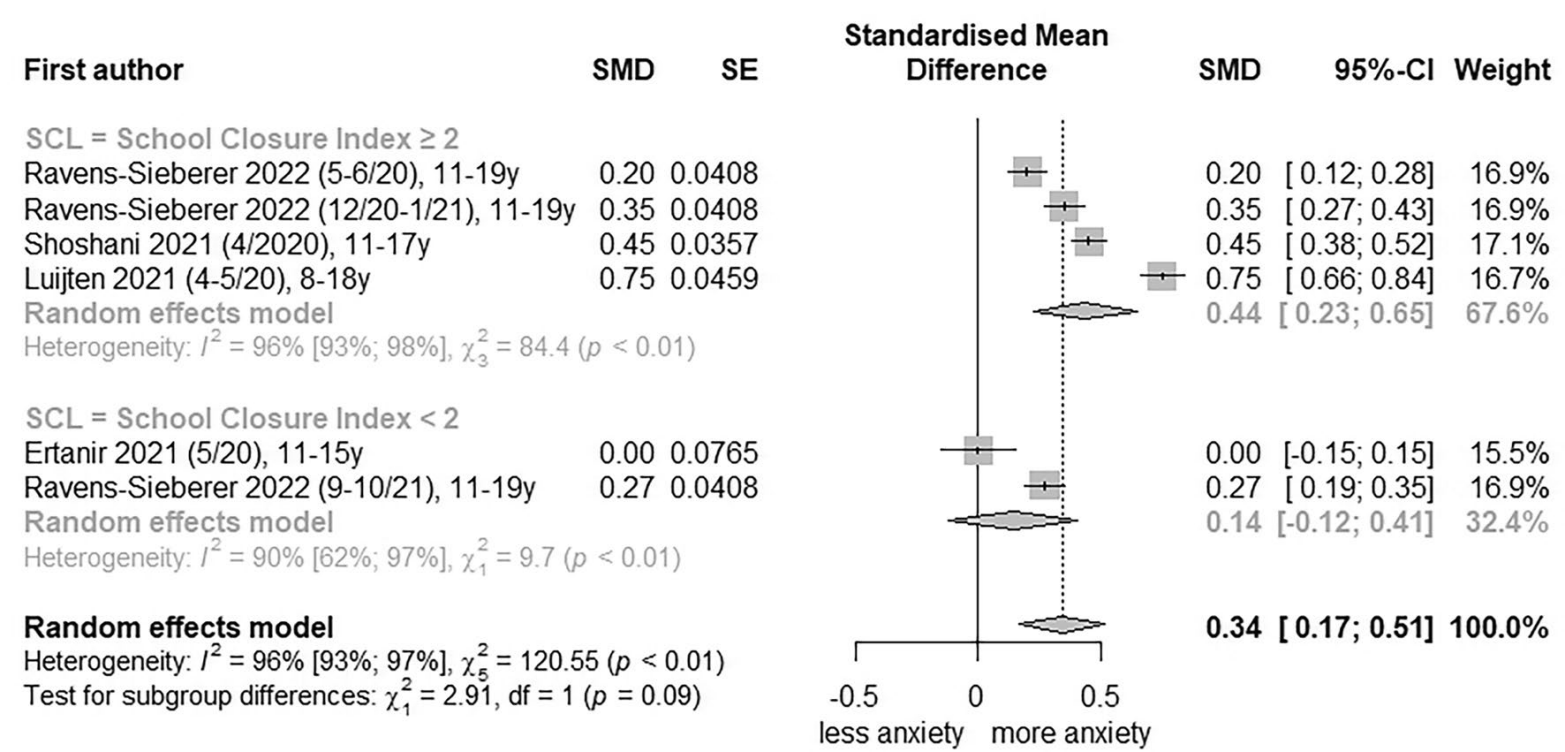
Forest plot of changes in youth general anxiety symptoms comparing School Closure Index. SE, standard error; SMD, standardized mean differences; SL, School Closure Index; 95%-CI, 95%-confidence interval

To estimate the extent to which the stringency of PHSM has an impact on anxiety symptoms, low RoB studies were pooled by the Oxford COVID-19 Stringency Index (> 60 vs ≤ 60) and the School Closure Index (≥ 2 and < 2). An increase in general anxiety symptoms was observed for the Oxford COVID-19 Stringency Index > 60 (SMD, 0.52; 95% CI, 0.30 to 0.73; I^2^ = 96%; Fig. 2) and the School Closure Index ≥ 2 (SMD, 0.44; 95% CI, 0.23 to 0.65; I^2^ = 96%; Fig. 3).

### Meta-analysis of clinically relevant anxiety rates

**Fig. 4:**
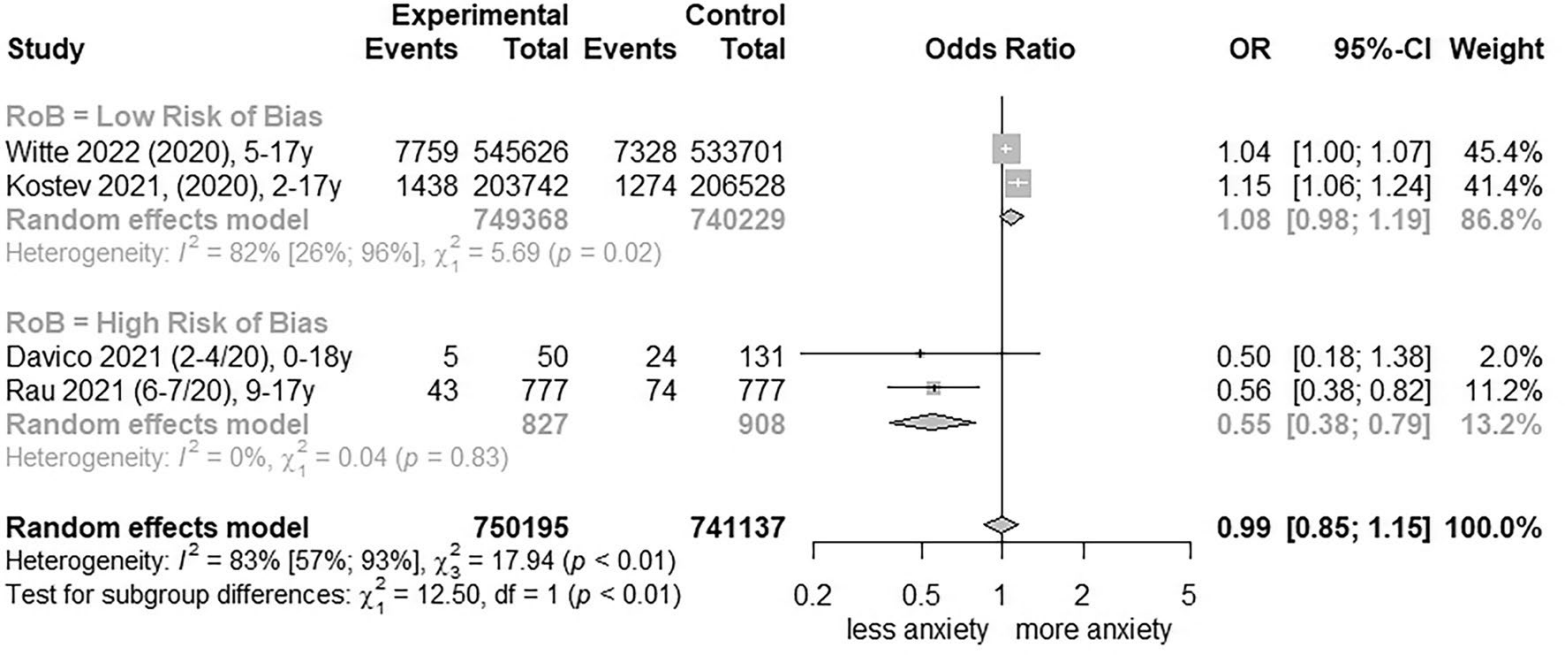
Forest plot of changes in youth clinically relevant anxiety symptoms comparing before and during COVID-19 pandemic. OR, Odds Ratio; RoB, risk of bias; 95%-CI, 95%-confidence interval

For clinically relevant anxiety rates, four studies [44, 45, 48, 56] were pooled and CoE was graded as ‘very low’ (Table 2; further information in Additional file 1: Table S7). Total change yielded an OR of 1.08 (95% CI, 0.98 to 1.19, I^2^ = 82%; Fig. 4) in two low RoB studies [44, 56]. Clinically relevant anxiety rates increased significantly in females in low RoB studies (OR, 1.10 [95% CI, 1.02 to 1.19], I^2^ = 52%; Additional file 1: Figure S12), but not for males (OR, 1.04 [95% CI, 0.92 to 1.17], I^2^ = 76%; Additional file 1: Figure S13).

### Heterogeneity, publication bias and sensitivity analysis

As heterogeneity was substantial in all meta-analyses (I^2^ > 50%), meta-regression analyses were conducted for the total population, female and male children and adolescents. In every meta-regression analysis, ‘RoB’ and ‘study design’ represent positive covariates (Additional file 1: Tables S9-14). The covariate ‘RoB’ was addressed by the aforementioned stratification of low vs high RoB studies. Effect direction and significance did not change after removing the study with cross-sectional design. Sensitivity analyses (Additional file 1: Table S15) revealed significant differences for study design and effect conversion. However, only one cross-sectional study and one study with converted measurements were included in the analyses. Effect direction and significance did not alter after removing these studies from meta-analyses. Visual analysis of the (contour-enhanced) funnel plots implied asymmetry (Additional file 1: Figures S14–S19), but was discarded by applying Egger’s test (Additional file 1: Table S16).

## Discussion

This systematic review and meta-analysis provides insights into the changes in general anxiety symptoms and clinically relevant anxiety rates in European children and adolescents after the onset of the COVID-19 pandemic when compared with the pre-pandemic baseline. We included 18 studies that assessed changes in over 750,000 children and adolescents (for several measurement points) across Europe. The pooled effect estimates of low RoB studies revealed an increase in general anxiety symptoms overall, and particularly for males in the 11–15 years age category. A significant increase in clinically relevant anxiety rates was also observed among female children and adolescents.

Considering the various different restriction policies in European countries, this systematic review and meta-analysis is the first that assessed the association between PHSM and higher general anxiety symptoms. For children above six years of age, school closures have been a major disruptor as these measures radically changed their life [9]. Instead of having social contact five days a week, often for six or eight hours a day with their class, peers and friends, they were homebound and unable to socialise properly. These full or partial school closures affected approximately 105 million pupils and students in Europe [57]. Our meta-analyses revealed particularly high general anxiety symptoms during periods of school closure (SMD, 0.44; 95% CI, 0.23 to 0.65) and other restriction measures (SMD, 0.52; 95% CI, 0.30 to 0.73); these effect increases outlined a potential impact of school closures and PHSM on anxiety symptoms. However, the evidence rating of "very low" have to be considered here; therefore, further reseach is needed. Both effect estimates were higher than in a previous meta-analysis on depression [31]. As social anxiety can be reduced through exposure to social interactions, the non-exposure to social contacts and social challenges in the school environment as a result of PHSM may explain the stronger correlation with the symptomatology. Further research will allow a comparison of the reduction in anxiety symptoms between subgroups and countries following the acute pandemic phase. Our results suggest that the higher association with restrictive measures could lead to a more rapid reduction in symptoms once life returns to normal. Nevertheless, social exclusion of children and adolescents during the pandemic could lead to life-long mental and physical health consequences [3, 58, 59]. However, a clearcut separation of the effects on anxiety due to school closure from those due to other pandemic related restrictions—like worries about (elderly) relatives, fear of long-lasting health effects (long COVID), and also closure of recreational and sports facilities—was not possible. This limitation was already found in a previous review [19]. Therefore, our results must be interpreted indicative regarding the possible drivers for the increased anxiety.

Regarding different subgroups, our analyses first showed strong differences between studies with low and high RoB. While the increase in general anxiety symptoms is clearly evident for studies with a low RoB, the pooling effects of high RoB studies were indistinct and non-significant. The heterogeneous evidence in literature can partly be attributed to the different quality of existing studies; this underlines the importance of strictly assessing the RoB. Second, age-specific analyses found considerably higher effect estimates for children and adolescents aged 11–15 years, in particular among males, but lower and more imprecise estimates for those aged 16–19 years. Taking into account the fact that the risk of anxiety disorders among children aged 10–14 years had already been reported as being high three decades before the COVID-19 pandemic [2], our findings showed that this age group was also more vulnerable to increases during the COVID-19 pandemic. The imprecise results for general anxiety symptoms among males in the 16–19 year age category are in contrast to findings on depression [31]. This underlines the necessity to differentiate between different mental health diagnoses in specific age groups in the COVID-19 pandemic. Third, for clinically relevant anxiety rates, the pooled associations were based on two low RoB studies from Germany and should be interpreted with caution; further empirical evidence is needed here.

This paper has strong implications for both policy and clinical practice. Policy-makers should consider the unintended consequences before imposing PHSM such as school closures on the mental health of children and adolescents. Psychiatrists, psychotherapists and other public health experts for children and adolescents should therefore be included in pandemic crisis task forces [60, 61]. The increase in general anxiety symptoms and the variation between specific groups and countries requires children and adolescents to be closely monitored over the next few years. This monitoring should cover a broad range of age groups, similar to the recommendation of the U.S. Preventive Services Task Force to screen all children and adolescents aged 8–18, regardless of whether they have symptoms [62]. Based on our study, children and adolescents born in 2005 to 2010 (aged 11–15 years in 2020 to 2021) should be monitored henceforth. While our study indicates a strong need for anxiety disorder therapies (like previous research for depression symptoms [31]), these professionals were understaffed even before the pandemic [6]. Policy makers should therefore strengthen availability and capacity of these professional groups.

Screening and adequate diagnoses are important for identifying children and adolescents with anxiety disorders and the need for therapy. The gap between studies measuring general anxiety symptoms and those measuring clinically relevant anxiety rates in our systematic review might indicate a lack of clinical evidence and diagnoses. Parents, teachers, health care professionals and sports trainers should be made aware of risk factors and symptoms of anxiety disorders as well as mental health services. The negative consequences in later life of a failure to address anxiety symptoms on children and adolescents are well documented [3, 58, 59]. Moreover, even before the COVID-19 pandemic, anxiety and depression disorders were two of the top five causes of overall disease burden for children and adolescents in Europe, and suicide was a leading cause of death among 10–19-year-olds in the WHO European region [3]. It is therefore important to implement evidence-based interventions that can help address mental health issues in children. Targeted interventions and longer programmes in particular seemed to be more effective [3]. In addition, protective factors should be communicated and supported; including parent–child dialogue [63], a predictable home environment [64], peer-to-peer social contact [65] and physical activity [66]. Further, increased resilience among child and adolescents could be a predictor of fewer anxiety symptoms [67, 68].

There are several research gaps regarding anxiety symptoms in the COVID-19 pandemic in Europe, including evidence for children aged below 10 years, differentiation by social status or education, and clinically relevant anxiety rates. Generally, there are only a very small number of studies on anxiety with a pre-pandemic baseline in Europe, although no such studies were able to be included for Eastern European countries and hardly any evidence from southern Europe. To improve this, representative longitudinal cohort or panel studies on CA should be conducted in all European countries so as to have a pre-crisis baseline and to monitor changes over time. Such a cohort or panel should include validated anxiety measures for general symptoms and for a clinically relevant cut-off, as well as demographic, socioeconomic and health-related confounders. These criteria are necessary in order to reduce the RoB and to allow subgroup-specific analyses.

### Strength and limitations

There are several limitations to this review. First, RoB was high for 12 studies (66% of the studies included), mainly based on bias due to participant selection, missing data and insufficient adjustment of important confounders. This limitation was addressed by downgrading for RoB in GRADE and we stratified our meta-analyses by RoB. Second, the instruments that were used differed greatly in their scales. To unify them, we transformed the effect estimates to SMD or OR. Third, there was a high level of heterogeneity in the meta-analyses (I^2^ > 50%), which we tried to explain by conducting meta-regression analyses. Fourth, no country pooling and visualisation over time were possible due to the low study quality. There were only a small number of available studies within our strict inclusion criteria with age-group-specific data. Fifth, there is a lack of longitudinal studies. Sixth, more subgroup analyses were not feasible. Seventh, the Oxford Stringency Index [8] and the School Closure Index [8] were used as proxies for PHSM and cannot cover all facets of the COVID-19 pandemic.

The strengths of this review are that it largely follows the methodological guidelines recommended by the Cochrane Handbook for systematic reviews [32], such as systematic search in several databases with a peer-reviewed search strategy and consideration of pre-prints, grey literature, and conference abstracts. In addition, literature screening, data extraction and RoB rating were performed independently and unpublished data was requested from study authors. In addition, the assessment of the RoB and the CoE was conducted using recommended tools. Thus, an assessment of evidence based on high quality studies was possible, allowing contradictory findings from previous studies to be properly interpreted.

## Conclusion

This systematic review and meta-analysis showed an increase in general anxiety symptoms among European children and adolescents during the first two years of the COVID-19 pandemics compared with a pre-pandemic baseline. The 11–15 years male age group was particularly affected. Social distancing policies implemented in European countries, and in particular school closures, might be associated with a considerable increase in the effect of general anxiety symptoms. Therefore, school closures should be implemented only with the greatest caution and with consideration of the evidence available regarding the mental health of children and adolescents. At present, the need is huge to monitor anxiety symptoms in children and adolescents on a long-term basis and to identify which of the 105 million children and adolescents in Europe have disorders that require professional management and treatment. Due to long-term consequences of anxiety disorders and the risk of suicidality, those affected have to be clinically addressed through early identification and therapy.

## Supplementary Information

**Additional file 1.** Additional Tables, Tables S1-S16 and additional Figures, Figures S1-S19.

## Data Availability

All data are included in the manuscript and appendix.
