# Supplementary material for: Anxiety increased among children and adolescents during pandemic-related school closures in Europe: a systematic review and meta-analysis
Source: Child Adolesc Psychiatry Ment Health. 2023 Jun 21;17:74. doi: 10.1186/s13034-023-00612-z (PMC10286360; doi:10.1186/s13034-023-00612-z)
Supplement: Supplementary file 1 — Additional file 1. Additional Tables, Tables S1-S16 and additional Figures, Figures S1-S19. [file 13034_2023_612_MOESM1_ESM.docx]

**Supplementary Information**

**Additional file 1.**

**Anxiety increased among children and adolescents during pandemic-related school closures in Europe: A systematic review and meta-analysis**

Helena Ludwig-Walz, PhD ^a^; Indra DANNHEIM, MA ^b,c^; Lisa M. Pfadenhauer, PhD ^d,e^; Jörg M. FEGERT, MD ^f^; Martin BUJARD, PhD ^g,f^

^a^ Federal Institute for Population Research (BiB), Wiesbaden, Germany

^b^ Regional Innovative Centre of Health and Quality of Live Fulda (RIGL), Fulda University of Applied Sciences, Fulda, Germany

^c^ Department of Nutritional, Food and Consumer Sciences, Fulda University of Applied Sciences, Fulda, Germany

^d^ Institute for Medical Information Processing, Biometry and Epidemiology - IBE, Chair of Public Health and Health Services Research, LMU Munich, Munich, Germany

^e^ Pettenkofer School of Public Health, Munich, Germany

^f^ University Medical Center, Department for Child and Adolescent Psychiatry and Psychotherapy, Competence Domain Mental Health Prevention, Ulm, Germany

^g^ Federal Institute for Population Research (BiB), Wiesbaden, Germany

^f^ Institute for Medical Psychology, Medical Faculty, University Heidelberg, Heidelberg, Germany

**Corresponding author:** Helena Ludwig-Walz, PhD, Federal Institute for Population Research (BiB), Wiesbaden, Germany ([helena.ludwig-walz@bib.bund.de](mailto:helena.ludwig-walz@bib.bund.de)).

Legend

[Table S1: PRISMA item checklist for systematic reviews 4](#_Toc136095846)

[Table S2: Deviations from the review protocol 7](#_Toc136095847)

[Table S3: Searched congresses and websites of key organizations 7](#_Toc136095848)

[Table S4: Search Strategy 8](#_Toc136095849)

[Table S5: Reasons for exclusion of studies from the systematic literature search, after full-text screening 14](#_Toc136095850)

[Table S6: Criteria for grading evidence according to Grading of Recommendations, Assessment, Development and Evaluations (GRADE) 15](#_Toc136095851)

[Table S7. Evidence profile for grading evidence according to Grading of Recommendations, Assessment, Development and Evaluations (GRADE) 18](#_Toc136095852)

[Table S8. Summary of effect estimates 19](#_Toc136095853)

[Table S9. Moderator analysis for total sample with categorical moderators 37](#_Toc136095854)

[Table S10. Moderator analysis for total sample with continuous moderators 37](#_Toc136095855)

[Table S11. Moderator analysis for female subsample with categorical moderators 37](#_Toc136095856)

[Table S12. Moderator analysis for female subsample with continuous moderators 37](#_Toc136095857)

[Table S13. Moderator analysis for male subsample with categorical moderators 38](#_Toc136095858)

[Table S14. Moderator analysis for male subsample with continuous moderators 38](#_Toc136095859)

[Table S15. Sensitivity analysis 38](#_Toc136095860)

[Table S16. Eggers’ test 38](#_Toc136095861)

[Figure S1: PRISMA Flow Chart 39](#_Toc136095862)

[Figure S2: Traffic-light plots of the domain-level judgements for each individual result 40](#_Toc136095863)

[Figure S3: Weighted-bar plots of the distribution of risk of bias judgements within each bias domain 41](#_Toc136095864)

[Figure S4: Forest Plot of Changes in Female General Anxiety Symptoms Comparing Before and During COVID-19 Pandemic 41](#_Toc136095865)

[Figure S5: Forest Plot of Changes in Male General Anxiety Symptoms Comparing Before and During COVID-19 Pandemic 41](#_Toc136095866)

[Figure S6. Forest Plot of Changes in Total (11-15 years) General Anxiety Symptoms Comparing Before and During COVID-19 Pandemic 42](#_Toc136095867)

[Figure S7. Forest Plot of Changes in Female (11-15 years) General Anxiety Symptoms Comparing Before and During COVID-19 Pandemic 42](#_Toc136095868)

[Figure S8. Forest Plot of Changes in Male (11-15 years) General Anxiety Symptoms Comparing Before and During COVID-19 Pandemic 42](#_Toc136095869)

[Figure S9. Forest Plot of Changes in Total (16-19 years) General Anxiety Symptoms Comparing Before and During COVID-19 Pandemic 43](#_Toc136095870)

[Figure S10. Forest Plot of Changes in Female (16-19 years) General Anxiety Symptoms Comparing Before and During COVID-19 Pandemic 43](#_Toc136095871)

[Figure S11. Forest Plot of Changes in Male (16-19 years) General Anxiety Symptoms Comparing Before and During COVID-19 Pandemic 43](#_Toc136095872)

[Figure S12. Forest Plot of Changes in Female Clinically Relevant Anxiety Rates Comparing Before and During COVID-19 Pandemic 44](#_Toc136095873)

[Figure S13. Forest Plot of Changes in Male Clinically Relevant Anxiety Rates Comparing Before and During COVID-19 Pandemic 44](#_Toc136095874)

[Figure S14. Funnel Plot of Changes in Total General Anxiety Symptoms Comparing Before and During COVID-19 Pandemic 45](#_Toc136095875)

[Figure S15. Funnel Plot of Changes in Female General Anxiety Symptoms Comparing Before and During COVID-19 Pandemic 46](#_Toc136095876)

[Figure S16. Funnel Plot of Changes in Male General Anxiety Symptoms Comparing Before and During COVID-19 Pandemic 47](#_Toc136095877)

[Figure S17. Funnel Plot of Changes in Total Clinically Relevant Anxiety Symptoms Comparing Before and During COVID-19 Pandemic 48](#_Toc136095878)

[Figure S18. Funnel Plot of Changes in Female Clinically Relevant Anxiety Symptoms Comparing Before and During COVID-19 Pandemic 49](#_Toc136095879)

[Figure S19. Funnel Plot of Changes in Male Clinically Relevant Anxiety Symptoms Comparing Before and During COVID-19 Pandemic 50](#_Toc136095880)

[References 51](#_Toc136095881)

# Table S1: PRISMA item checklist for systematic reviews

| **Topic** | **No.** | **Item** | **Location where item is reported** |
| --- | --- | --- | --- |
| **TITLE** |  |  |  |
| **Title** | 1 | Identify the report as a systematic review. | Title |
| **ABSTRACT** |  |  |  |
| **Abstract** | 2 | See the PRISMA 2020 for Abstracts checklist | not applicable |
| **INTRODUCTION** |  |  |  |
| **Rationale** | 3 | Describe the rationale for the review in the context of existing knowledge. | Background |
| **Objectives** | 4 | Provide an explicit statement of the objective(s) or question(s) the review addresses. | Background |
| **METHODS** |  |  |  |
| **Eligibility criteria** | 5 | Specify the inclusion and exclusion criteria for the review and how studies were grouped for the syntheses. | Methods/Search strategy and selection criteria |
| **Information sources** | 6 | Specify all databases, registers, websites, organisations, reference lists and other sources searched or consulted to identify studies. Specify the date when each source was last searched or consulted. | Methods/Search strategy and selection criteria |
| **Search strategy** | 7 | Present the full search strategies for all databases, registers and websites, including any filters and limits used. | eTable 4 |
| **Selection process** | 8 | Specify the methods used to decide whether a study met the inclusion criteria of the review, including how many reviewers screened each record and each report retrieved, whether they worked independently, and if applicable, details of automation tools used in the process. | Methods/Search strategy and selection criteria |
| **Data collection process** | 9 | Specify the methods used to collect data from reports, including how many reviewers collected data from each report, whether they worked independently, any processes for obtaining or confirming data from study investigators, and if applicable, details of automation tools used in the process. | Methods/Data analysis |
| **Data items** | 10a | List and define all outcomes for which data were sought. Specify whether all results that were compatible with each outcome domain in each study were sought (e.g. for all measures, time points, analyses), and if not, the methods used to decide which results to collect. | Methods/Data analysis |
|  | 10b | List and define all other variables for which data were sought (e.g. participant and intervention characteristics, funding sources). Describe any assumptions made about any missing or unclear information. | Methods/Data analysis |
| **Study risk of bias assessment** | 11 | Specify the methods used to assess risk of bias in the included studies, including details of the tool(s) used, how many reviewers assessed each study and whether they worked independently, and if applicable, details of automation tools used in the process. | Methods/Data analysis |
| **Effect measures** | 12 | Specify for each outcome the effect measure(s) (e.g. risk ratio, mean difference) used in the synthesis or presentation of results. | Methods/Data analysis |
| **Synthesis methods** | 13a | Describe the processes used to decide which studies were eligible for each synthesis (e.g. tabulating the study intervention characteristics and comparing against the planned groups for each synthesis (item 5)). | Methods/Data analysis |
|  | 13b | Describe any methods required to prepare the data for presentation or synthesis, such as handling of missing summary statistics, or data conversions. | Methods/Data analysis |
|  | 13c | Describe any methods used to tabulate or visually display results of individual studies and syntheses. | Methods/Data analysis |
|  | 13d | Describe any methods used to synthesize results and provide a rationale for the choice(s). If meta-analysis was performed, describe the model(s), method(s) to identify the presence and extent of statistical heterogeneity, and software package(s) used. | Methods/Data analysis |
|  | 13e | Describe any methods used to explore possible causes of heterogeneity among study results (e.g. subgroup analysis, meta-regression). | Methods/Data analysis |
|  | 13f | Describe any sensitivity analyses conducted to assess robustness of the synthesized results. | Methods/Data analysis |
| **Reporting bias assessment** | 14 | Describe any methods used to assess risk of bias due to missing results in a synthesis (arising from reporting biases). | Methods/Data analysis |
| **Certainty assessment** | 15 | Describe any methods used to assess certainty (or confidence) in the body of evidence for an outcome. | Methods/Data analysis |
| **RESULTS** |  |  |  |
| **Study selection** | 16a | Describe the results of the search and selection process, from the number of records identified in the search to the number of studies included in the review, ideally using a flow diagram. | Results, eFigure 1 |
|  | 16b | Cite studies that might appear to meet the inclusion criteria, but which were excluded, and explain why they were excluded. | eTable 5 |
| **Study characteristics** | 17 | Cite each included study and present its characteristics. | Table 1 |
| **Risk of bias in studies** | 18 | Present assessments of risk of bias for each included study. | eFigures 2 and 3 |
| **Results of individual studies** | 19 | For all outcomes, present, for each study: (a) summary statistics for each group (where appropriate) and (b) an effect estimate and its precision (e.g. confidence/credible interval), ideally using structured tables or plots. | eTable 8 |
| **Results of syntheses** | 20a | For each synthesis, briefly summarise the characteristics and risk of bias among contributing studies. | Results, Figures 1-4 |
|  | 20b | Present results of all statistical syntheses conducted. If meta-analysis was done, present for each the summary estimate and its precision (e.g. confidence/credible interval) and measures of statistical heterogeneity. If comparing groups, describe the direction of the effect. | Results,  Figures 1-4, eFigures 4-13 |
|  | 20c | Present results of all investigations of possible causes of heterogeneity among study results. | Results, eFigures 9-15 |
|  | 20d | Present results of all sensitivity analyses conducted to assess the robustness of the synthesized results. | Results,  eTable 15 |
| **Reporting biases** | 21 | Present assessments of risk of bias due to missing results (arising from reporting biases) for each synthesis assessed. | Results,  eTable 16, eFigures 14-19 |
| **Certainty of evidence** | 22 | Present assessments of certainty (or confidence) in the body of evidence for each outcome assessed. | Results, eTables 6 and 7 |
| **DISCUSSION** |  |  |  |
| **Discussion** | 23a | Provide a general interpretation of the results in the context of other evidence. | Discussion |
|  | 23b | Discuss any limitations of the evidence included in the review. | Discussion |
|  | 23c | Discuss any limitations of the review processes used. | Discussion |
|  | 23d | Discuss implications of the results for practice, policy, and future research. | Discussion |
| **OTHER INFORMATION** |  |  |  |
| **Registration and protocol** | 24a | Provide registration information for the review, including register name and registration number, or state that the review was not registered. | Methods/Search strategy and selection criteria |
|  | 24b | Indicate where the review protocol can be accessed, or state that a protocol was not prepared. | Methods/Search strategy and selection criteria |
|  | 24c | Describe and explain any amendments to information provided at registration or in the protocol. | Methods/Search strategy and selection criteria |
| **Support** | 25 | Describe sources of financial or non-financial support for the review, and the role of the funders or sponsors in the review. | not applicable |
| **Competing interests** | 26 | Declare any competing interests of review authors. | Competing interests |
| **Availability of data, code and other materials** | 27 | Report which of the following are publicly available and where they can be found: template data collection forms; data extracted from included studies; data used for all analyses; analytic code; any other materials used in the review. | Additional information (online) |

# Table S2: Deviations from the review protocol

- Differentiation of clinically relevant anxiety rates according to the Oxford COVID-19 Stringency Index and School Closure Index was not possible due to an insufficient number of included low risk of bias studies.
- No subgroup analyses could be conducted regarding social status or education, due to a lack of information in the included studies.
- All discrepancies regarding literature screening, risk of bias assessment and GRADE assessment could be resolved without integration of a third author.
- Because of the differentiated research questions, the Joanna Briggs Institute (JBI) Manual for Evidence Synthesis could not be applied.

# Table S3: Searched congresses and websites of key organizations

| **Congresses:** | - European Public Health Conference - European Conference on Mental Health |
| --- | --- |
| **Websites of key organizations:** | - European Centre for Disease Prevention and Control - European Society for Child and Adolescent Psychiatry - European Union - Council of Europe - Save the Children - United Nations International Children's Emergency Fund (UNICEF) - World Health Organization (WHO) - Organization for Economic Co-operation and Development (OECD) |

# Table S4: Search Strategy

**PubMed**

| **Population** | Infan* OR toddler* OR minors OR minors* OR boy OR boys OR boyfriend OR boyhood OR girl* OR kid OR kids OR child OR child* OR children* OR schoolchild* OR schoolchild OR school child[tiab] OR school child*[tiab] OR adolescen* OR juvenil* OR youth* OR teen* OR under*age* OR pubescen* OR pediatrics[mh] OR pediatric* OR paediatric* OR peadiatric* OR school*[tiab] OR kindergarten*[tiab] OR kindergarden*[tiab] |
| --- | --- |
| **Intervention** | ("Covid-19"[Mesh] OR covid*[tiab] OR "SARS-CoV-2"[Mesh] OR “sars-2”[tiab] OR “sars2”[tiab] OR “sars 2”[tiab] OR “sars-cov-19”[tiab] OR “sars-cov19”[tiab] OR “sarscov-19”[tiab] OR “sarscov19”[tiab] OR “sars cov 19”[tiab] OR “sarscov 19”[tiab] OR “sars cov19”[tiab] OR "sars-cov-2"[tiab] OR “sarscov-2”[tiab] OR “sars-cov2”[tiab] OR “sarscov2”[tiab] OR "sars cov 2"[tiab] OR "sarscov 2"[tiab] OR "sars cov2"[tiab] OR "Severe Acute Respiratory Syndrome" [tiab] OR "Severe Acute Respiratory disease"[tiab] OR "coronavirus"[MeSH] OR Coronavirus*[tiab] OR corona-virus*[tiab] OR “corona virus*”[tiab] OR ncov*[tiab] OR n-cov*[tiab] OR “n cov”[tiab] OR novelcov*[tiab] OR novel-cov*[tiab] OR “novel cov*”[tiab]) AND (2019/11/01[PDAT] : 3000/12/31[PDAT]) |
| **Comparison** |  |
| **Outcome** | “Anxiety”[Mesh] OR “Anxiety Disorders”[Mesh] OR anxi*[tiab] |
| **Species** | FinalResult NOT (animals[MeSH] NOT humans[MeSH]) |
| **Language** | No limit |

**Embase**

| **Population** | Infan* OR toddler* OR minors* OR boy OR boys OR boyfriend OR boyhood OR girl* OR kid OR kids OR child* OR schoolchild* OR (‘school child’ OR ‘school child*’):ab,ti OR adolescen* OR juvenil* OR youth* OR teen* OR under*age* OR pubescen* OR 'pediatrics'/exp OR pediatric* OR paediatric* OR (‘peadiatric*’ OR ‘school’ OR ‘school*’):ab,ti OR (kindergarten* OR kindergarden*):ab,ti |
| --- | --- |
| **Intervention** | (covid*:ab,ti OR 'sars 2':ab,ti OR sars2:ab,ti OR 'sars-2':ab,ti OR 'sars-cov-19':ab,ti OR 'sars-cov19':ab,ti OR 'sarscov-19':ab,ti OR 'sarscov19':ab,ti OR 'sars cov 19':ab,ti OR 'sarscov 19':ab,ti OR 'sars cov19':ab,ti OR 'sars-cov-2':ab,ti OR 'sarscov-2':ab,ti OR 'sars-cov2':ab,ti OR 'sarscov2':ab,ti OR 'sars cov 2':ab,ti OR 'sarscov 2':ab,ti OR 'sars cov2':ab,ti OR 'severe acute respiratory syndrome':ab,ti OR 'severe acute respiratory disease':ab,ti OR coronavirus*:ab,ti OR 'corona virus*':ab,ti OR 'corona-virus*':ab,ti OR ncov*:ab,ti OR 'n-cov*':ab,ti OR 'n cov':ab,ti OR novelcov*:ab,ti OR 'novel cov*':ab,ti OR 'novel-cov*':ab,ti OR 'severe acute respiratory syndrome coronavirus 2'/exp OR 'coronavirinae'/exp OR 'coronavirus'/exp) AND [01-11-2019]/sd NOT [31-12-3000]/sd |
| **Comparison** |  |
| **Outcome** | 'anxiety'/exp OR 'anxiety disorder'/exp OR 'anxi*':ab,ti |
| **Species** | FinalResult NOT ([animals]/lim NOT [humans]/lim) |
| **Language** | No limit |

**APA PsycInfo (via EBSCOhost)**

| **Population** | (TI (Infan* OR toddler* OR minors OR minors* OR boy OR boys OR boyfriend OR boyhood OR girl* OR kid OR kids OR child OR child* OR children* OR schoolchild* OR schoolchild OR ‘school child’ OR ‘school child*’ OR adolescen* OR juvenil* OR youth* OR teen* OR underage* OR pubescen* OR pediatrics OR pediatric* OR paediatric* OR ‘school*’ OR kindergarten* OR kindergarden*)) OR (AB (Infan* OR toddler* OR minors OR minors* OR boy OR boys OR boyfriend OR boyhood OR girl* OR kid OR kids OR child OR child* OR children* OR schoolchild* OR schoolchild OR ‘school child’ OR ‘school child*’ OR adolescen* OR juvenil* OR youth* OR teen* OR underage* OR pubescen* OR pediatrics OR pediatric* OR paediatric* OR ‘school*’ OR kindergarten* OR kindergarden*)) |
| --- | --- |
| **Intervention** | (DE "COVID-19" OR DE "Coronavirus") OR (TI covid* OR AB covid*) OR (TI covid* OR AB covid*) OR (TI "SARS-CoV-2" OR AB "SARS-CoV-2") OR (TI "sars-2*" OR AB "sars-2*") OR (TI "sars2*" OR AB "sars2*") OR (TI "sars 2" OR AB "sars 2") OR (TI “sars-cov-19” OR AB “sars-cov-19”) OR (TI “sars-cov19” OR AB “sars-cov19”) OR (TI “sarscov-19” OR AB “sarscov-19”) OR (TI “sarscov19” OR AB “sarscov19”) OR (TI “sars cov 19” OR AB “sars cov 19”) OR (TI “sarscov 19” OR AB “sarscov 19”) OR (TI “sars cov19” OR AB “sars cov19”) OR (TI “sarscov-2” OR AB “sarscov-2”) OR (TI “sars-cov-2” OR AB “sars-cov-2”) OR (TI “sarscov2” OR AB “sarscov2”) OR (TI “sars-cov2” OR AB “sars-cov2”) OR (TI “sarscov2” OR AB “sarscov2”) OR (TI “sars cov 2” OR AB “sars cov 2”) OR (TI “sarscov 2” OR AB “sarscov 2”) OR (TI “sars cov2” OR AB “sars cov2”) (TI “Severe Acute Respiratory Syndrome” OR AB “Severe Acute Respiratory Syndrome”) OR (TI “Severe Acute Respiratory disease” OR AB “Severe Acute Respiratory disease”) OR (TI “Coronavirus*” OR AB “Coronavirus*”) OR (TI “Corona-virus*” OR AB “Corona-virus*”) OR (TI “Corona virus*” OR AB “Corona virus*”) OR (TI “ncov*” OR AB “ncov*”) OR (TI “n-cov*” OR AB “n-cov*”) OR (TI “n cov*” OR AB “n cov*”) OR (TI “novelcov*” OR AB “novelcov*”) OR (TI “novel-cov*” OR AB “novel-cov*”) OR (TI “novel cov*” OR AB “novel cov*”) |
| **Comparison** |  |
| **Outcome** | (DE "Anxiety") OR (DE "Anxiety Disorders") OR (TI(anxi*)) OR (AB(anxi*)) |
| **Limits** | Filters: Humans *(screened manually)* |

**Cochrane Library**

| **Population** | #1 MeSH descriptor: [Infant] explode all trees  #2 MeSH descriptor: [Child] explode all trees  #3 MeSH descriptor: [Adolescent] explode all trees  #4 MeSH descriptor: [Pediatrics] explode all trees  #5 MeSH descriptor: [Schools] explode all trees  #6 (Infan* OR toddler* OR minors OR minors* OR boy OR boys OR boyfriend OR boyhood OR girl* OR kid OR kids OR child* OR schoolchild* OR schoolchild OR "school NEXT child*" OR adolescen* OR juvenil* OR youth* OR teen* OR under-age* OR pubescen* OR paediatric* OR peadiatric* OR school* OR kindergarten* OR kindergarden*):ti,kw,ab |
| --- | --- |
| **Intervention** | #7 MeSH descriptor: [COVID-19] explode all trees  #8 MeSH descriptor: [Coronavirus] explode all trees  #9 MeSH descriptor: [SARS-CoV-2] explode all trees  #10 (covid* OR sars-2 OR sars2 OR "sars NEXT 2" OR sars-cov-19 OR sars-cov19 OR sarscov-19 OR “sars NEXT cov NEXT 19” OR “sarscov NEXT 19” OR “sars NEXT cov19” OR sarscov19 OR SARS-CoV-2 OR sarscov-2 OR sars-cov2 OR sarscov2 OR "sars NEXT cov NEXT 2" OR "sarscov NEXT 2" OR "sars NEXT cov2" OR "Severe NEXT Acute NEXT Respiratory NEXT Syndrome" OR "Severe NEXT Acute NEXT Respiratory NEXT disease" OR Coronavirus* OR corona-virus* OR "corona NEXT virus*" OR ncov* OR n-cov* OR "n NEXT cov" OR novelcov* OR novel-cov* OR "novel NEXT cov*"):ti,kw,ab |
| **Comparison** |  |
| **Outcome** | #11 MeSH descriptor: [Anxiety] explode all trees 8823  #12 MeSH descriptor: [Anxiety Disorders] explode all trees 7539  #13 (anxi*):ti,kw,ab |
| **Limits** | Filters: Humans *(screened manually)* |

**Web of Science Core Collection**

| **Population** | ts=(Infan* OR toddler* OR minors* OR boy OR boys OR boyfriend OR boyhood OR girl* OR kid OR kids OR child* OR children* OR schoolchild* OR schoolchild OR "school child" OR "school child" OR adolescen* OR juvenil* OR youth* OR teen* OR under*age* OR pubescen* OR kindergarten* OR kindergarden*) |
| --- | --- |
| **Intervention** | ts=(covid* OR "sars 2" OR sars2 OR sars-2 OR "sars cov 19" OR "sars cov19" OR "sarscov 19" OR sarscov19 OR "sars-cov-19" OR "sars-cov19" OR "sarscov-19" OR "sars-cov-2" OR "sarscov 2" OR "sars cov2" OR sarscov2 OR "sars cov 2" OR "sarscov-2" OR "sars-cov2" OR "severe acute respiratory syndrome" OR "severe acute respiratory disease" OR coronavirus* OR "corona virus*" OR "corona-virus*" OR ncov* OR "n-cov*" OR "n cov" OR novelcov* OR "novel cov*" OR "novel-cov*") |
| **Comparison** | NA |
| **Outcome** | ts=(anxi*) |
| **Species** | Humans *(screened manually)* |
| **Language** | No limit |

**WHO COVID-19 database**

| **Population** | (Infan* toddler* OR minors* OR boy OR boys OR boyfriend OR boyhood OR girl* OR kid OR kids OR child* OR children* OR schoolchild* OR schoolchild OR "school child" OR "school child" OR adolescen* OR juvenil* OR youth* OR teen* OR under*age* OR pubescen* OR kindergarten* OR kindergarden*) |
| --- | --- |
| **Intervention** | (covid* OR "sars 2" OR sars2 OR sars-2 OR "sars cov 19" OR "sars cov19" OR "sarscov 19" OR sarscov19 OR "sars-cov-19" OR "sars-cov19" OR "sarscov-19" OR "sars-cov-2" OR "sarscov 2" OR "sars cov2" OR sarscov2 OR "sars cov 2" OR "sarscov-2" OR "sars-cov2" OR "severe acute respiratory syndrome" OR "severe acute respiratory disease" OR coronavirus* OR "corona virus*" OR "corona-virus*" OR ncov* OR "n-cov*" OR "n cov" OR novelcov* OR "novel cov*" OR "novel-cov*") |
| **Comparison** | NA |
| **Outcome** | (anxi*) |
| **Species** | Humans *(screened manually)* |
| **Language** | No limit |

# Table S5: Reasons for exclusion of studies from the systematic literature search, after full-text screening

| **Study** | **Reason for exclusion** |
| --- | --- |
| Achterberg et al. 2021 | No data on anxiety reported. |
| Albrecht et al. 2022 | No pre-pandemic baseline was reported. |
| Allgaier et al. 2022 | No data on anxiety reported. |
| Borbas et al. 2020 | No data on anxiety reported. |
| Bujard et al. 2021 | No data on anxiety reported. |
| Burdzovic et al. 2021 | No data on anxiety reported. |
| Daniunaite et al. 2021 | No data on anxiety reported. |
| Dollberg et al. 2021 | No data on anxiety reported. |
| Dragun et al. 2020 | Inappropriate study population (>19 years of age). |
| Duttweiler et al. 2022 | Inappropriate study population (USA). |
| Dzielska et al. 2021 | No data on anxiety reported. |
| Elise et al. 2021 | No data on anxiety reported. |
| Essau et al. 2021 | No pre-pandemic baseline was reported. |
| Gonzalez-Valero et al. 2020 | No data on anxiety reported. |
| Halldorsdottir et al. 2021 | No data on anxiety reported. |
| Hoffmann et al. 2021 | Inappropriate study population (>19 years of age). |
| Janssen et al. 2021 | No data on anxiety reported. |
| Koenig et al. 2021 | Inappropriate study population (age range 12-20 years of age). |
| Larsen et al. 2021 | No pre-pandemic baseline was reported. |
| Mohler-Kuo et al. 2021 | No pre-pandemic baseline was reported. |
| Muzi et al. 2021 | No data on anxiety reported. |
| Myhr et al. 2021 | No data on anxiety reported. |
| Naumann et al. 2021 | Same study population as in Bujard et al. 2021 [4]. |
| Newlove-Delgado et al. 2021 | No data on anxiety reported. |
| Ravens-Sieberer et al. 2021 [5] | Same study population as in Ravens-Sieberer et al. 2022 [6]. |
| Ravens-Sieberer et al. 2021 [7] | Same study population as in Ravens-Sieberer et al. 2022 [6]. |
| Ravens-Sieberer et al. 2021 [8] | Same study population as in Ravens-Sieberer et al. 2022[6]. |
| Soest et al. 2021 | No data on anxiety reported. |
| Thorisdottir et al. 2021 | No data on anxiety reported. |
| Truskauskaite-Kuneviciene et al. 2021 | Inappropriate study population (>19 years of age). |
| Ünver et al. 2022 | Inappropriate study population (Syrian refugee). |
| van der Laan et al. 2021 | No data on anxiety reported. |
| Vogel et al. 2021 | No data on anxiety reported. |

# Table S6: Criteria for grading evidence according to Grading of Recommendations, Assessment, Development and Evaluations (GRADE)

Developed under consideration of the criteria reported by Schünemann et al. 2013 [2], Schünemann et al. 2019 [3] and Morgan et al. 2019 [1].

| **Domain** | **Application in this review** | **Interpretation** | **GRADE assessment** |
| --- | --- | --- | --- |
| *Reasons for considering lowering certainty* | |  |  |
| Risk of Bias (RoB) | Based on the RoB assessment using the RoB instrument for non-randomized studies of exposures [1]. | 1. Most information is from results at low risk of bias. 2. Most information is from results at low risk of bias or with some concerns; potential limitations are unlikely to lower confidence in the estimate of effect. 3. Most information is from results at low risk of bias or with some concerns; potential limitations are likely to lower confidence in the estimate of effect. 4. The proportion of information from results at high risk of bias is sufficient to affect the interpretation of results; crucial limitation for one criterion, or some limitations for multiple criteria, sufficient to lower confidence in the estimate of effect. 5. The proportion of information from results at high risk of bias is sufficient to affect the interpretation of results; crucial limitation for one or more criteria sufficient to substantially lower confidence in the estimate of effect. | 1. No serious limitations, not downgrade. 2. No serious limitations, not downgrade. 3. Serious limitations, downgrade 1 level. 4. Serious limitations, downgrade 1 level. 5. Very serious limitations, downgrade 2 levels. |
| Inconsistency | Existence of heterogeneity, if   1. Wide variance of point estimates across studies 2. Minimal or no overlap of confidence intervals (CI) 3. Statistical criteria: chi^2^ test and I² statistic    - Significant p-value in chi² test 🡪 indication of heterogeneity    - I² statistic    - < 40% 🡪 heterogeneity may be low    - 30-60% 🡪 heterogeneity may be moderate    - 50-90% 🡪 heterogeneity may be substantial    - 75-100% 🡪 heterogeneity may be considerable | - - - 1. No wide variance of point estimates, minimal or no overlap of CI, chi² test not significant,  I² <60%.       2. Chi² test significant, I² test >50%, further analysis via subgroup analysis, sensitivity analysis, meta-regression analysis.       3. Chi² test significant, I² test >50%, no further analysis. | 1. No serious limitations, not downgrade. 2. Serious limitations, downgrade 1 level. 3. Very serious limitations, downgrade 2 levels. |
| Indirectness | Applicability of the evidence to the relevant research question. Fitting of the characteristics of the included studies to the predefined PECO scheme. | - - - 1. Studies address exactly the research question.       2. Studies indirectly (indirect comparisons) or imprecisely (restricted version of the main review question) address the research question, but an adequate transfer is possible.       3. Studies indirectly (indirect comparisons) or imprecisely (restricted version of the main review question) address the research question, but an adequate transfer is not possible. | 1. No serious limitations, not downgrade. 2. Serious limitations, downgrade 1 level. 3. Very serious limitations, downgrade 2 levels. |
| Imprecision | Risks of random errors because of small sample sizes, broad CIs or inclusion of the “no effect line” in 95% CI | - - - 1. Broad sample size with small to moderate confidence intervals and no overlap of the “no effect line” (mean/SMD=0, OR/RR=1) regarding the 95% CI of the pooled effect.       2. Moderate to broad sample size, with moderate confidence intervals and overlap of the line of no effect of the 95% CI.       3. Small sample size, with moderate to broad confidence intervals and overlap of the line of no effect of the 95% CI. | 1. No serious limitations, not downgrade. 2. Serious limitations, downgrade 1 level. 3. Very serious limitations, downgrade 2 levels. |
| Publication bias | Under-estimation or over-estimation of the underlying beneficial or harmful effect due to the selective publication of studies if   - - Asymmetrical funnel plot   - Significant Egger’s Test when at least 10 studies | 1. No funnel plot asymmetry and no significance in Egger’s Test. 2. Funnel plot asymmetry and/or significance in Egger’s Test. | 1. No serious limitations, not downgrade. 2. Serious limitations, downgrade 1 level. |
|  |  |  |  |
| *Reasons for considering upgrade certainty* | |  |  |
| Large effect | Rating up when effects in observational studies are sufficiently large, particularly if they occur over short periods of time. | 1. RR >2 or RR <0.5 2. RR >5 or RR <0.2   *Note from the GRADE Handbook [2]: these rules apply when effect measure is expressed as relative risk (RR) or hazard ratio (HR). They cannot always be applied when the effect measure is expressed as odds ratio (OR). We suggest converting OR to RR and only then assessing the magnitude of an effect.* | - - - 1. May upgrade 1 level.       2. May upgrade 2 levels. |
| Dose-response | Rating up for a dose-response gradient if effect estimates are higher when pandemic related restrictions are rigorous, measured by the proxy variables “Oxford COVID-19 Stringency Index” and the “School Closure Index”. | 1. No/small differences in pandemic-related restrictions and no substantial subgroup differences. 2. Effect estimates for severe pandemic-related restrictions higher with substantial subgroup differences to moderate/lead restrictions. | 1. No upgrade. 2. May upgrade 1 level. |
| All plausible confounding and bias | All plausible residual confounding from observational studies may be working to reduce the demonstrated effect or increase the effect, if no effect was observed. | 1. No underestimation of an apparent treatment effect. 2. Underestimate of an apparent treatment effect. | 1. No upgrade. 2. May upgrade 1 level. |
|  |  |  |  |
| **Certainty of evidence** | **High – Moderate – Low – Very low** | | |

# Table S7. Evidence profile for grading evidence according to Grading of Recommendations, Assessment, Development and Evaluations (GRADE)

# Table S8. Summary of effect estimates

| **STUDY INFO** | **REPORTED Effect estimate** | | | **(converted) effect estimate*** | | | | **Risk of bias** |
| --- | --- | --- | --- | --- | --- | --- | --- | --- |
| **First author, year, country** | **Effect measure**  **Subsamples** | **Adjustment** | **Reported outcome** | **During pandemic estimate, n/N or mean (SD) N** | **Pre-pandemic estimate, n/N or mean (SD) N** | **Std. Mean difference  (95% CI)** | **Odds ratio  (95% CI)** |  |
| **Germany** |  |  |  |  |  |  |  |  |
| Ravens-Sieberer, 2022 [6] | Screen for Child Anxiety Related Disorders (SCARED) | No adjustment | General anxiety symptoms  *Additional data were provided by authors* | **Total (11-19 y):**  DP1: 5.66 (4.28) 1,018  DP2: 6.27 (4.53) 1,073  DP3: 5.94 (4.49) 1,173  **Female (11-19 y):**  DP1: 6.26 (4.52) 505  DP2: 6.86 (4.62) 528  DP3: 6.48 (4.68) 568  **Male (11-19 y):**  DP1: 5.01 (3.91) 513  DP2: 5.70 (4.38) 545  DP3: 5.39 (4.20) 597  **Total (11-14 y):**  DP1: 5.91 (4.37) 572  DP2: 6.21 (4.53) 536  DP3: 5.98 (4.40) 528  **Female (11-14 y):**  DP1: 6.33 (4.68) 291  DP2: 6.32 (4.54) 255  DP3: 6.31 (4.63) 260  **Male (11-14 y):**  DP1: 5.43 (3.94) 280  DP2: 6.09 (4.50) 281  DP3: 5.67 (4.12) 263  **Total (15-19 y):**  DP1: 5.33 (4.15) 446  DP2: 6.34 (4.54) 536  DP3: 5.91 (4.57) 645  **Female (15-19 y):**  DP1: 6.23 (4.31) 213  DP2: 7.36 (4.63) 273  DP3: 6.63 (4.73) 308  **Male (15-19 y):**  DP1: 4.51 (3.83) 233  DP2: 5.28 (4.20) 263  DP3: 5.17 (4.25) 334 | **Total (11-19 y):**  4.87 (3.50) 1,333  **Female (11-19 y):**  5.62 (3.56) 630  **Male (11-19 y):**  4.20 (3.30) 703  **Total (11-14 y):**  4.53 (3.45) 807  **Female (11-14 y):**  5.17 (3.47) 380  **Male (11-14 y):**  3.95 (3.34) 427  **Total (15-19 y):**  5.40 (3.51) 526  **Female (15-19 y):**  6.30 (3.60) 250  **Male (15-19 y):**  4.59 (3.22) 276 | **Total (11-19 y):**  DP1: 0.20 (0.12 to 0.28)³  DP2: 0.35 (0.27 to 0.43)³  DP3: 0.27 (0.19 to 0.35)³  **Female (11-19 y):**  DP1: 0.16 (0.04 to 0.28)³  DP2: 0.30 (0.19 to 0.41)³  DP3: 0.21 (0.09 to 0.33)³  **Male (11-19 y):**  DP1: 0.23 (0.11 to 0.35)³  DP2: 0.39 (0.28 to 0.50)³  DP3: 0.32 (0.21 to 0.43)³  **Total (11-14 y):**  DP1: 0.36 (0.25 to 0.47)³  DP2: 0.38 (0.27 to 0.49)³  DP3: 0.43 (0.32 to 0.54)³  **Female (11-14 y):**  DP1: 0.29 (0.13 to 0.45)³  DP2: 0.29 (0.13 to 0.45)³  DP3: 0.29 (0.13 to 0.45)³  **Male (11-14 y):**  DP1: 0.41 (0.26 to 0.56)³  DP2: 0.56 (0.40 to 0.72)³  DP3: 0.47 (0.31 to 0.63)³  **Total (15-19 y):**  DP1: -0.02 (-0.14 to 0.10)³  DP2: 0.23 (0.11 to 0.35)³  DP3: 0.13 (-0.03 to 0.29)³  **Female (15-19 y):**  DP1: -0.02 (-0.20 to 0.16)³  DP2: 0.25 (0.08 to 0.42)³  DP3: 0.08 (-0.09 to 0.25)³  **Male (15-19 y):**  DP1: -0.02 (-0.20 to 0.16)³  DP2: 0.18 (0.02 to 0.34)³  DP3: 0.16 (-0.01 to 0.33)³ |  | **Mode-rate** |
|  |  |  | Cut off≥9 SCARED | **Total (11-14 y):**  DP1: 147/572  DP2: 152/536  DP3: 148/528  **Female (11-14 y):**  DP1: 88/291  DP2: 73/255  DP3: 83/260  **Male (11-14 y):**  DP1: 58/280  DP2: 79/281  DP3: 65/199  **Total (15-19 y):**  DP1: 99/446  DP2: 154/471  DP3: 130/476  **Female (15-19 y):**  DP1: 62/213  DP2: 107/240  DP3: 85/145  **Male (15-19 y):**  DP1: 37/233  DP2: 47/231  DP3: 42/200  **Total (11-19 y):**  DP1: 246/1,018  DP2: 322/1,073  DP3: 314/1,137  **Female (11-19 y):**  DP1: 150/505  DP2: 190/528  DP3: 181/568  **Male (11-19 y):**  DP1: 95/513  DP2: 132/545  DP3: 129/597 | **Total (11-14 y):**  101/807  **Female (11-14 y):**  62/380  **Male (11-14 y):**  39/427  **Total (15-19 y):**  97/526  **Female (15-19 y):**  64/250  **Male (15-19 y):**  33/276  **Total (11-19 y):**  198/1,333  **Female (11-19 y):**  126/630  **Male (11-19 y):**  72/703 |  | **Total (11-14 y):**  DP1: 2.42 (1.83 to 3.20)³  DP2: 2.77 (2.09 to 3.66)³  DP3: 2.72 (2.05 to 3.61)³  **Female (11-14 y):**  DP1: 2.22 (1.54 to 3.22)³  DP2: 2.06 (1.40 to 3.02)³  DP3: 2.41 (1.65 to 3.51)³  **Male (11-14 y):**  DP1: 2.60 (1.68 to 4.03)³  DP2: 3.89 (2.56 to 5.92)³  DP3: 4.83 (3.10 to 7.51)³  **Total (15-19 y):**  DP1: 1.26 (0.92 to 1.73)³  DP2: 2.15 (1.60 to 2.88)³  DP3: 1.66 (1.23 to 2.24)³  **Female (15-19 y):**  DP1: 1.19 (0.79 to 1.80)³  DP2: 2.34 (1.60 to 3.42)³  DP3: 4.12 (2.66 to 6.37)³  **Male (15-19 y):**  DP1: 1.39 (0.84 to 2.30)³  DP2: 1.88 (1.16 to 3.05)³  DP3: 1.96 (1.19 to 3.22)³  **Total (11-19 y):**  DP1: 1.83 (1.48 to 2.25)³  DP2: 2.46 (2.01 to 3.00)³  DP3: 2.19 (1.79 to 2.67)³  **Female (11-19 y):**  DP1: 1.69 (1.29 to 2.22)³  DP2: 2.25 (1.73 to 2.93)³  DP3: 1.87 (1.44 to 2.43)³  **Male (11-19 y):**  DP1: 1.99 (1.43 to 2.77)³  DP2: 2.80 (2.05 to 3.83)³  DP3: 2.42 (1.77 to 3.30)³ |  |
| Witte,  2022 [9] | Medical classification (anxiety disorder diagnoses [ICD-10: F40/41])  Subgroups:   - Gender - Age | No adjustment | Clinically relevant anxiety symptoms  *Additional data were provided by authors* | **Total (5-17 y), prevalence:**  11,674/545,626  **Total (5-17 y), incidence:**  7,759/545,626  **Female (5-17 y), prevalence:**  7,191/265,213  **Female (5-17 y), incidence:**  4,865/265,213  **Male (5-17 y), prevalence:**  4,483/280,413  **Male (5-17 y), incidence:**  2,894/280,413  **Total (5-9 y), prevalence:**  2,305/200,054  **Total (5-9 y), incidence:**  1,812/200,054  **Female (5-9 y), prevalence:**  1,183/97,523  **Female (5-9 y), incidence:**  922/97,523  **Male (5-9 y), prevalence:**  1,122/102,531  **Male (5-9 y), incidence:**  890/102,531  **Total (10-14 y), prevalence:**  4,271/212,447  **Total (10-14 y), incidence:**  2,758/212,447  **Female (10-14 y), prevalence:**  2,403/102,980  **Female (10-14 y), incidence:**  1,628/102,980  **Male (10-14 y), prevalence:**  1,868/109,467  **Male (10-14 y), incidence:**  1,130/109,467  **Total (15-17 y), prevalence:**  5,098/133,125  **Total (15-17 y), incidence:**  3,189/133,125  **Female (15-17 y), prevalence:**  3,605/64,710  **Female (15-17 y), incidence:**  2,315/64,710  **Male (15-17 y), prevalence:**  1,493/68,415  **Male (15-17 y), incidence:**  874/68,415  **Total (10-17 y), stationary care DP1:**  281/339,361  **Female (10-17 y), stationary care DP1:**  182/164,829  **Male (10-17 y), stationary care DP1:**  99/174,532  **Total (10-14 y), stationary care DP1:**  99/209,495  **Female (10-14 y), stationary care DP1:**  58/101,584  **Male (10-14 y), stationary care DP1:**  41/107,911  **Total (15-17 y), stationary care DP1:**  182/129,866  **Female (15-17 y), stationary care DP1:**  124/63,245  **Male (15-17 y), stationary care DP1:**  58/66,621  **Total (10-17 y), stationary care DP2:**  315/343,642  **Female (10-17 y), stationary care DP2:**  238/166,707  **Male (10-17 y), stationary care DP2:**  77/176,935  **Total (10-14 y), stationary care DP2:**  125/211,881  **Female (10-14 y), stationary care DP2:**  95/102,804  **Male (10-14 y), stationary care DP2:**  30/109,077  **Total (15-17 y), stationary care DP2:**  190/131,761  **Female (15-17 y), stationary care DP2:**  143/63,903  **Male (15-17 y), stationary care DP2:**  47/67,858 | **Total (5-17 y), prevalence:**  11,008/533,701  **Total (5-17 y), incidence:**  7,328/533,701  **Female (5-17 y), prevalence:**  6,631/259,455  **Female (5-17 y), incidence:**  4,446/259,455  **Male (5-17 y), prevalence:**  4,377/274,246  **Male (5-17 y), incidence:**  2,882/274,246  **Total (5-9 y), prevalence:**  2,364/194,120  **Total (5-9 y), incidence:**  1,861/194,120  **Female (5-9 y), prevalence:**  1,212/94,492  **Female (5-9 y), incidence:**  968/94,492  **Male (5-9 y), prevalence:**  1,152/99,628  **Male (5-9 y), incidence:**  893/99,628  **Total (10-14 y), prevalence:**  4,097/208,420  **Total (10-14 y), incidence:**  2,697/208,420  **Female (10-14 y), prevalence:**  2,302/100,972  **Female (10-14 y), incidence:**  1,572/100,972  **Male (10-14 y), prevalence:**  1,795/107,448  **Male (10-14 y), incidence:**  1,125/107,448  **Total (15-17 y), prevalence:**  4,547/131,161  **Total (15-17 y), incidence:**  2,770/131,161  **Female (15-17 y), prevalence:**  3,117/63,991  **Female (15-17 y), incidence:**  1,906/63,991  **Male (15-17 y), prevalence:**  1,430/67,170  **Male (15-17 y), incidence:**  864/67,170  **Total (10-17 y), stationary care DP1:**  297/332,945  **Female (10-17 y), stationary care DP1:**  206/161,957  **Male (10-17 y), stationary care DP1:**  91/170,988  **Total (10-14 y), stationary care DP1:**  108/205,399  **Female (10-14 y), stationary care DP1:**  70/99,551  **Male (10-14 y), stationary care DP1:**  38/105,848  **Total (15-17 y), stationary care DP1:**  189/127,546  **Female (15-17 y), stationary care DP1:**  136/62,406  **Male (15-17 y), stationary care DP1:**  53/65,140  **Total (10-17 y), stationary care DP2:**  1,342/332,945  **Female (10-17 y), stationary care DP2:**  297/161,957  **Male (10-17 y), stationary care DP2:**  91/170,988  **Total (10-14 y), stationary care DP2:**  108/205,399  **Female (10-14 y), stationary care DP2:**  70/99,551  **Male (10-14 y), stationary care DP2:**  38/105,848  **Total (15-17 y), stationary care DP2:**  297/127,546  **Female (15-17 y), stationary care DP2:**  206/62,406  **Male (15-17 y), stationary care DP2:**  91/65,140 |  | **Total (5-17 y),  prevalence:**  1.04 (1.01 to 1.07)³  **Total (5-17 y),  incidence:**  1.04 (1.00 to 1.07)³  **Female (5-17 y),**  **prevalence:**  1.06 (1.03 to 1.10)³  **Female (5-17 y),**  **incidence:**  1.07 (1.03 to 1.12)³  **Male (5-17 y),  prevalence:**  1.00 (0.96 to 1.04)³  **Male (5-17 y),  incidence:**  0.98 (0.93 to 1.03)³  **Total (5-9 y),  prevalence:**  0.95 (0.89 to 1.00)³  **Total (5-9 y),  incidence:**  0.94 (0.88 to 1.01)³  **Female (5-9 y),  prevalence:**  0.95 (0.87 to 1.02)³  **Female (5-9 y),  incidence:**  0.92 (0.84 to 1.01)³  **Male (5-9 y),  prevalence:**  0.95 (0.87 to 1.03)³  **Male (5-9 y),  incidence:**  0.97 (0.88 to 1.06)³  **Total (10-14 y),**  **prevalence:**  1.02 (0.98 to 1.07)³  **Total (10-14 y),  incidence:**  1.00 (0.95 to 1.06)³  **Female (10-14 y), prevalence:**  1.02 (0.97 to 1.09)³  **Female (10-14 y),**  **incidence:**  1.02 (0.95 to 1.09)³  **Male (10-14 y),  prevalence:**  1.02 (0.96 to 1.09)³  **Male (10-14 y),  incidence:**  0.99 (0.91 to 1.07)³  **Total (15-17 y),**  **prevalence:**  1.11 (1.06 to 1.15)³  **Total (15-17 y),  incidence:**  1.14 (1.08 to 1.20)³  **Female (15-17 y), prevalence:**  1.15 (1.10 to 1.21)³  **Female (15-17 y),  incidence:**  1.21 (1.14 to 1.29)³  **Male (15-17 y),**  **prevalence:**  1.03 (0.95 to 1.10)³  **Male (15-17 y),  incidence:**  0.99 (0.90 to 1.09)³  **Total (10-17 y), stationary care PP -> DP1:**  0.93 (0.79 to 1.09)³  **Female (10-17 y), stationary care PP -> DP1:**  0.87 (0.71 to 1.06)³  **Male (10-17 y), stationary care PP -> DP1:**  1.07 (0.80 to 1.42)³  **Total (10-14 y), stationary care PP -> DP1:**  0.90 (0.68 to 1.18)³  **Female (10-14 y), stationary care PP -> DP1:**  0.81 (0.57 to 1.15)³  **Male (10-14 y), stationary care PP -> DP1:**  1.06 (0.68 to 1.65)³  **Total (15-17 y), stationary care PP -> DP1:**  0.95 (0.77 to 1.16)³  **Female (15-17 y), stationary care PP -> DP1:**  0.90 (0.70 to 1.15)³  **Male (15-17 y), stationary care PP -> DP1:**  1.07 (0.74 to 1.55)³  **Total (10-17 y), stationary care PP -> DP2:**  1.03 (0.88 to 1.20)³  **Female (10-17 y), stationary care PP -> DP2:**  1.12 (0.93 to 1.35)³  **Male (10-17 y), stationary care PP -> DP2:**  0.82 (0.60 to 1.11)³  **Total (10-14 y), stationary care PP -> DP2:**  1.12 (0.87 to 1.45)³  **Female (10-14 y), stationary care PP -> DP2:**  1.31 (0.97 to 1.79)³  **Male (10-14 y), stationary care PP -> DP2:**  0.77 (0.47 to 1.24)³  **Total (15-17 y), stationary care PP -> DP2:**  0.97 (0.80 to 1.19)³  **Female (15-17 y), stationary care PP -> DP2:**  1.03 (0.81 to 1.30)³  **Male (15-17 y), stationary care PP -> DP2:**  0.85 (0.57 to 1.26)³ | **Mode-rate** |
| Kostev,  2021 [10] | Medical classification (anxiety disorder diagnoses [ICD-10: F41])  Subgroups:   - Gender - Age | No adjustment | Clinically relevant anxiety rates  *Additional data were provided by authors* | **Total (2-17 y), prevalence:**  1,861/203,742  **Total (2-17 y), incidence:**  1,438/203,742  **Female (2-17 y), prevalence:**  1,037/80,964  **Female (2-17 y), incidence:**  798/80,964  **Male (2-17 y), prevalence:**  824/122,778  **Male (2-17 y), incidence:**  640/122,778  **Total (2-5 y), prevalence:**  387/107,629  **Total (2-5 y), incidence:**  320/107,629  **Female (2-5 y), prevalence:**  213/46,024  **Female (2-5 y), incidence:**  171/46,024  **Male (2-5 y), prevalence:**  174/61,605  **Male (2-5 y), incidence:**  149/61,605  **Total (6-9 y), prevalence:**  434/44,545  **Total (6-9 y), incidence:**  347/44,545  **Female (6-9 y), prevalence:**  208/15,378  **Female (6-9 y), incidence:**  169/15,378  **Male (6-9 y), prevalence:**  226/29,167  **Male (6-9 y), incidence:**  178/29,167  **Total (10-12 y), prevalence:**  441/23,986  **Total (10-12 y), incidence:**  346/23,986  **Female (10-12 y), prevalence:**  219/7,908  **Female (10-12 y), incidence:**  169/7,908  **Male (10-12 y), prevalence:**  222/16,078  **Male (10-12 y), incidence:**  177/16,078  **Total (13-17 y), prevalence:**  599/27,582  **Total (13-17 y), incidence:**  425/27,582  **Female (13-17 y), prevalence:**  397/11,654  **Female (13-17 y), incidence:**  289/11,654  **Male (13-17 y), prevalence:**  202/15,928  **Male (13-17 y), incidence:**  136/15,928 | **Total (2-17 y), prevalence:**  1,701/206,528  **Total (2-17 y), incidence:**  1,274/206,528  **Female (2-17 y), prevalence:**  916/80,934  **Female (2-17 y), incidence:**  687/80,934  **Male (2-17 y), prevalence:**  785/125,594  **Male (2-17 y), incidence:**  587/125,594  **Total (2-5 y), prevalence:**  388/108,204  **Total (2-5 y), incidence:**  314/108,204  **Female (2-5 y), prevalence:**  189/46,313  **Female (2-5 y), incidence:**  146/46,313  **Male (2-5 y), prevalence:**  199/61,891  **Male (2-5 y), incidence:**  168/61,891  **Total (6-9 y), prevalence:**  383/45,570  **Total (6-9 y), incidence:**  300/45,570  **Female (6-9 y), prevalence:**  203/15,659  **Female (6-9 y), incidence:**  162/15,659  **Male (6-9 y), prevalence:**  180/29,911  **Male (6-9 y), incidence:**  138/29,911  **Total (10-12 y), prevalence:**  379/24,797  **Total (10-12 y), incidence:**  276/24,797  **Female (10-12 y), prevalence:**  176/8,046  **Female (10-12 y), incidence:**  136/8,046  **Male (10-12 y), prevalence:**  203/16,751  **Male (10-12 y), incidence:**  140/16,751  **Total (13-17 y), prevalence:**  551/27,957  **Total (13-17 y), incidence:**  384/27,957  **Female (13-17 y), prevalence:**  348/10,916  **Female (13-17 y), incidence:**  243/10,916  **Male (13-17 y), prevalence:**  203/17,041  **Male (13-17 y), incidence:**  141/17,041 |  | **Total (2-17 y),  prevalence:**  1.11 (1.04 to 1.19)³  **Total (2-17 y),  incidence:**  1.15 (1.06 to 1.24)³  **Female (2-17 y),  prevalence:**  1.13 (1.04 to 1.24)³  **Female (2-17 y),  incidence:**  1.16 (1.05 to 1.29)³  **Male (2-17 y),  prevalence:**  1.07 (0.97 to 1.18)³  **Male (2-17 y),  incidence:**  1.12 (1.00 to 1.25)³  **Total (2-5 y),  prevalence:**  1.00 (0.87 to 1.15)³  **Total (2-5 y),  incidence:**  1.02 (0.88 to 1.20)³  **Female (2-5 y),  prevalence:**  1.13 (0.93 to 1.38)³  **Female (2-5 y),  incidence:**  1.18 (0.95 to 1.47)³  **Male (2-5 y),  prevalence:**  0.88 (0.72 to 1.08)³  **Male (2-5 y),  incidence:**  0.89 (0.71 to 1.11)³  **Total (6-9 y),  prevalence:**  1.16 (1.01 to 1.33)³  **Total (6-9 y),  incidence:**  1.18 (1.01 to 1.38)³  **Female (6-9 y),  prevalence:**  1.04 (0.86 to 1.27)³  **Female (6-9 y),  incidence:**  1.06 (0.86 to 1.32)³  **Male (6-9 y),  prevalence:**  1.29 (1.06 to 1.57)³  **Male (6-9 y),  incidence:**  1.32 (1.06 to 1.66)³  **Total (10-12 y),  prevalence:**  1.21 (1.05 to 1.39)³  **Total (10-12 y),  incidence:**  1.30 (1.11 to 1.52)³  **Female (10-12 y), prevalence:**  1.27 (1.04 to 1.56)³  **Female (10-12 y),  incidence:**  1.27 (1.01 to 1.60)³  **Male (10-12 y),  prevalence:**  1.14 (0.94 to 1.38)³  **Male (10-12 y),  incidence:**  1.32 (1.06 to 1.65)³  **Total (13-17 y),  prevalence:**  1.10 (0.98 to 1.24)³  **Total (13-17 y),  incidence:**  1.12 (0.98 to 1.29³  **Female (13-17 y), prevalence:**  1.07 (0.93 to 1.24)³  **Female (13-17 y),  incidence:**  1.12 (0.94 to 1.33)³  **Male (13-17 y),  prevalence:**  1.07 (0.88 to 1.30)³  **Male (13-17 y),  incidence:**  1.03 (0.81 to 1.31)³ | **Mode-rate** |
| Rau, 2021 [11] | Revised Child Anxiety and Depression Scale (RCADS)  Subgroups:   - Gender | No adjustment | General anxiety symptoms  *Additional data were provided by authors* | **Total:**  21.1 (17.0) 777  **Female:**  26.6 (18.6) 414  **Male:**  14.9 (12.4) 363 | **Total:**  PP1:  24.4 (17.7) 777  PP2:  25.1 (19.1) 777  **Female:**  PP1:  29.6 (19.1) 414  PP2:  30.8 (20.0) 414  **Male:**  PP1:  18.5 (13.6) 363  PP2:  18.7 (15.6) 363 | **Total:**  PP1 -> DP: -0.19 (-0.29 to -0.09)  PP2 -> DP:  -0.19 (-0.29 to -0.09)  **Female:**  PP1 -> DP:  -0.16 (-0.30 to -0.02)  PP2 -> DP:  -0.22 (-0.35 to -0.09)  **Male:**  PP1 -> DP:  -0.28 (-0.42 to -0.14)  PP2 -> DP:  -0.27 (-0.42 to -0.12) |  | **Serious** |
|  |  |  | Clinically relevant anxiety rates,  clinical cut-off:  T-Score>65 | **Total (9-17 y):**  43/777  **Female (9-17 y):**  36/414  **Male (9-17 y):**  7/363 | **Total (9-17 y):**  PP1: 59/777  PP2: 74/777  **Female (9-17 y):**  PP1: 48/414  PP2: 55/414  **Male (9-17 y):**  PP1: 11/363  PP2: 19/363 |  | **Total (9-17 y):**  PP1 -> DP:  0.71 (0.47 to 1.07)³  PP2 -> DP:  0.56 (0.38 to 0.82)³  **Female (9-17 y):**  PP1 -> DP:  0.73 (0.46 to 1.15)³  PP2 -> DP:  0.62 (0.40 to 0.97)³  **Male (9-17 y):**  PP1 -> DP:  0.63 (0.24 to 1.64)^3^  PP2 -> DP:  0.36 (0.15 to 0.86)³ |  |
| **Israel** |  |  |  |  |  |  |  |  |
| Shoshani, 2021 [12] | Brief Symptom Inventory 18 (BSI-18), subscale anxiety | No adjustment | General anxiety symptoms  *Additional data were requested from the authors; no data were provided* | **Total (11-17 y):**  5.24 (3.14) 1,537 | **Total (11-17 y):**  3.93 (2.68) 1,537 | **Total (11-17 y):**  0.45 (0.38 to 0.52)^1^ |  | **Mode-rate** |
| **Italy** |  |  |  |  |  |  |  |  |
| Frigerio, 2022 [13] | Child Behavior Checklist (CBCL 1½-5), subscale anxious/ depressed  Subgroups:   - Gender | No adjustment | General anxiety symptoms  *Additional data were not usable* | **Total (1-5 y):**  2.85 (2.22) 59  **Female (1-5 y):**  2.33 (1.96) 27  **Male (1-5 y):**  3.28 (2.36) 32 | **Total (1-5 y):**  PP1:  1.83 (1.60) 94  PP2:  1.89 (1.74) 88  **Female (1-5 y):**  PP1:  1.75 (1.70) 44  PP2:  1.54 (1.85) 41  **Male (1-5 y):**  PP1:  1.90 (1.51) 50  PP2:  2.19 (1.61) 47 | **Total (1-5 y):**  PP1 -> DP:  0.54 (0.21 to 0.88)^1^  PP2 -> DP:  0.49 (0.16 to 0.83)^1^  **Female (1-5 y):**  PP1 -> DP:  0.32 (-0.16 to 0.80)^1^  PP2 -> DP:  0.41 (-0.08 to 0.90)^1^  **Male (1-5 y):**  PP1 -> DP:  0.73 (0.27 to 1.18)^1^  PP2 -> DP:  0.55 (0.10 to 1.01)^1^ |  | **Serious** |
| Davico, 2021 [14] | Psychiatric Emergency Department visits | No adjustment | Clinically relevant anxiety symptoms  *Additional data were requested from the authors; no data were provided* | **Total (0-18 y):**  5/50  *Data estimated from Figure 3* | **Total (0-18 y):**  PP1: 11/101  PP2: 21/93  PP3: 24/131  *Data estimated from Figure 3* |  | **Total (0-18 y):**  PP3 -> DP:  0.50 (0.18 to 1.38) | **Critical** |
| Crescentini 2020 [15] | Child Behavior Checklist (CBCL 6-18), subscale anxiety | No adjustment | General anxiety symptoms  Effect of Time (not reported here)  *Additional data were provided by authors* | **Total (6-18 y):**  5.15 (3.95) 721  **Female (6-18 y):**  5.21 (3.90) 349  **Male (6-18 y):**  5.08 (3.99) 372  **Total (6-10y):**  5.32 (4.06) 419  **Female (6-10y):**  5.38 (3.95) 197  **Male (6-10y):**  5.26 (4.15) 222  **Total (11-15y):**  4.95 (3.85) 288  **Female (11-15y):**  4.99 (3.82) 148  **Male (11-15y):**  4.91 (3.80) 140  **Total (16-18y):**  3.93 (2.63) 14  **Female (16-18y):**  5.00 (3.00) 4  **Male (16-18y):**  3.50 (2.33) 10 | **Total (6-18 y):**  4.56 (3.71) 721  **Female (6-18 y):**  4.52 (3.58) 349  **Male (6-18 y):**  4.60 (3.58) 372  **Total (6-10y):**  4.46 (3.58) 419  **Female (6-10y):**  4.40 (3.44) 197  **Male (6-10y):**  4.50 (3.70) 222  **Total (11-15y):**  4.74 (3.91) 288  **Female (11-15y):**  4.68 (3.77) 148  **Male (11-15y):**  4.80 (4.05) 140  **Total (16-18y):**  4.21 (3.03) 14  **Female (16-18y):**  4.50 (2.87) 4  **Male (16-18y):**  4.10 (3.08) 10 | **Total (6-18 y):**  0.15 (0.05 to 0.25)^1^  **Female (6-18 y):**  0.18 (0.04 to 0.32)^3^  **Male (6-18 y):**  0.12 (-0.02 to 0.26)^3^  **Total (6-10y):**  0.22 (0.09 to 0.35)^3^  **Female (6-10y):**  0.26 (0.07 to 0.45)^3^  **Male (6-10y):**  0.19 (0.01 to 0.37)^3^  **Total (11-15y):**  0.05 (-0.11 to 0.21)^3^  **Female (11-15y):**  0.08 (-0.15 to 0.31)^3^  **Male (11-15y):**  0.03 (-0.21 to 0.27)^3^  **Total (16-18y):**  -0.10 (-0.84 to 0.64)^3^  **Female (16-18y):**  0.15 (-1.24 to 1.54)^3^  **Male (16-18y):**  -0.21 (-1.09 to 0.67)^3^ |  | **Critical** |
| **Netherlands** | |  |  |  |  |  |  |  |
| Luijten, 2021 [16] | Patient-Reported Outcome Measurement Information System (PROMIS), CAT V2.0-Anxiety | No adjustment | General anxiety symptoms  *Additional data were provided by authors* | **Total (8-18 y):**  50.5 (7.6) 813  **Female (8-18 y):**  50.7 (7.4) 444  **Male (8-18 y):**  50.4 (7.8) 400  **Total (8-10 y):**  51.4 (8.0) 195  **Female (8-10 y):**  51.5 (8.4) 111  **Male (8-10 y):**  51.3 (7.6) 84  **Total (11-15 y):**  50.7 (7.6) 431  **Female (11-15 y):**  50.5 (7.3) 224  **Male (11-15 y):**  51.0 (7.9) 207  **Total (16-18 y):**  49.0 (7.2) 189  **Female (16-18 y):**  50.3 (6.9) 99  **Male (16-18 y):**  47.5 (6.9) 90 | **Total (8-18 y):**  43.8 (9.7) 1,318  **Female (8-18 y):**  44.8 (10.0) 667  **Male (8-18 y):**  42.7 (9.6) 652  **Total (8-10 y):**  44.5 (9.6) 394  **Female (8-10 y):**  44.1 (9.4) 204  **Male (8-10 y):**  45.0 (9.8) 190  **Total (11-15 y):**  43.7 (9.8) 618  **Female (11-15 y):**  44.8 (10.0) 289  **Male (11-15 y):**  42.7 (9.5) 329  **Total (16-18 y):**  43.0 (10.4) 307  **Female (16-18 y):**  45.7 (10.7) 174  **Male (16-18 y):**  39.5 (8.9) 133 | **Total (8-18 y):**  0.75 (0.66 to 0.84)^1^  **Female (8-18 y):**  0.65 (0.53 to 0.77)³  **Male (8-18 y):**  0.86 (0.73 to 0.99)³  **Total (8-10 y):**  0.76 (0.58 to 0.94)³  **Female (8-10 y):**  0.81 (0.57 to 1.05)³  **Male (8-10 y):**  0.68 (0.42 to 0.94)³  **Total (11-15 y):**  0.78 (0.65 to 0.91)³  **Female (11-15 y):**  0.64 (0.46 to 0.82)³  **Male (11-15 y):**  0.93 (0.75 to 1.11)³  **Total (16-18 y):**  0.64 (0.46 to 0.82)³  **Female (16-18 y):**  0.48 (0.23 to 0.73)³  **Male (16-18 y):**  0.98 (0.70 to 1.26)³ |  | **Mode-rate** |
|  |  |  | Cut off > 1.5 SD on PROMIS domains (severe symptoms) | **Participants with poor functioning or severe symptoms**  136/813 | **Participants with poor functioning or severe symptoms**  113/1,318 |  | **Participants with poor functioning or severe symptoms**  2.14 (1.64 to 2.80)^1^ |  |
| **Norway** |  |  |  |  |  |  |  |  |
| Hafstad, 2021 [17] | HSCL-10, anxiety/depression symptoms | No adjustment | General anxiety symptoms  *Additional data were requested from the authors; no data were provided* | **Total (12-16y):**  0.57 (0.64) 3,572 (estimated)  **Total (12-16y), clinical cut:**  196/3,572 | **Total (12-16y):**  0.51 (0.62) 3,572*  (estimated)  **Total (12-16y), clinical cut:**  225/3,572*  **inconsistent reporting in original study* | **Total (12-16y):**  0.10 (0.05 to 0.14)^1^ | **Total (12-16y), clinical cut:**  0.86 (0.71 to 1.05)^1^ | **Serious** |
| **Spain** |  |  |  |  |  |  |  |  |
| Giménez-Dasí, 2021 [18] | Anxiety scale “System of Evaluation of Children and Adolescents” (SENA) questionnaire  Subgroup:   - Gender | No adjustment | General anxiety symptoms  *Additional data were provided by authors* | **Total (6-11y):**  DP1: 2.15 (0.68) 66  DP2: 2.20 (0.74) 205  **Female (6-11y):**  DP1: 2.04 (0.68) 41  DP2: 2.23 (0.82) 110  **Male (6-11y):**  DP1: 2.34 (0.65) 25  DP2: 2.17 (0.62) 95 | **Total (6-11y):**  2.37 (0.66) 206  **Female (6-11y):**  2.36 (0.62) 107  **Male (6-11y):**  2.38 (0.70) 99 | **Total (6-11y):**  DP1: -0.33 (-0.61 to -0.05)³  DP2: -0.24 (-0.44 to -0.04)³  **Female (6-11y):**  DP1: -0.50 (-0.86 to -0.14)³  DP2: -0.18 (-0.44 to 0.08)³  **Male (6-11y):**  DP1: -0.06 (-0.50 to 0.38)³  DP2: -0.32 (-0.60 to -0.04)³ |  | **Serious** |
| Carrillo-Diaz, 2022 [19] | State Anxiety Scale (STAI-S) | No adjustment | General anxiety symptoms  *Additional data were requested from the authors; no data were provided* | **Total (11-17 y):**  32.7 (13.4) 213 | **Total (11-17 y):**  18 (10.3) 213 | **Total (11-17 y):**  1.23 (1.02 to 1.44)^1^ |  | **Critical** |
| **Switzerland** |  |  |  |  |  |  |  |  |
| Ertanir, 2021 [20] | Hopkins Symptoms Checklist (HSCL-25), subscale anxiety  Subgroups:   - Gender | No adjustment | General anxiety symptoms  *Additional data were provided by authors* | **Total (11-15 y):**  1.95 (0.64) 315  **Female (11-15 y):**  2.15 (0.65) 134  **Male (11-15 y):** 1.77 (0.58) 163 | **Total (11-15 y):**  1.95 (0.62) 357  **Female (11-15 y):**  2.04 (0.67) 166  **Male (11-15 y):**  1.86 (0.57) 185 | **Total (11-15 y):**  0.00 (-0.15 to 0.15)³  **Female (11-15 y):**  0.17 (-0.06 to 0.40)³  **Male (11-15 y):**  -0.16 (-0.37 to 0.05)³ |  | **Mode-rate** |
| Borbás, 2021 [21] | Child Behavior Checklist (CBCL 6-18), subscales anxious/ depressed | No adjustment | General anxiety symptoms  *No further data were requested.* | **Total (7-17 y):**  55 (8.32) 26 | **Total (7-17 y):**  56.73 (8.49) 26 | **Total (7-17y):**  -0.20 (-0.75 to 0.34)^1^ |  | **Critical** |
| **United Kingdom** | |  |  |  |  |  |  |  |
| Widnall, 2022 [22] | Hospital Anxiety & Depression Scale (HADS)  Subgroups:   - Gender | No adjustment | General anxiety symptoms  *Additional data were provided by authors* | **Total (13-15 y):**  DP1: 6.87 (4.74) 587  DP2: 7.99 (5.01) 589  **Female (13-15 y):**  DP1: 8.17 (4.75) 345  DP2: 9.71 (4.83) 352  **Male (13-15 y):**  DP1: 4.93 (4.93) 237  DP2: 5.41 (4.09) 232 | **Total (13-15 y):**  8.00 (4.70) 588  **Female (13-15 y):**  9.10 (4.90) 349  **Male (13-15 y):**  6.35 (3.85) 235 | **Total (13-15 y):**  DP1: -0.24 (-0.35 to -0.13)^3^  DP2: -0.00 (-0.12 to 0.12)^3^  **Female (13-15 y):**  DP1: -0.19 (-0.34 to -0.04)^3^  DP2: 0.13 (-0.02 to 0.28)^3^  **Male (13-15 y):**  DP1: -0.32 (-0.50 to 0.14)^3^  DP2: -0.24 (-0.35 to -0.13)^3^ | | **Mode-rate** |
| Knowles,  2022 [23] | Generalized Anxiety Disorder Scale (GAD-7)  Subsamples:   - Gender | No adjustment | General anxiety symptoms (Cut off ≥10 GAD-7)  *Additional data were requested from the authors; no data were provided* | **Total (12-18 y):**  185/1,069 (weighted)  **Female (12-18 y):**  135/584^b^  (weighted)  **Male (12-18 y):**  50/490^b^ (weighted)  *^b^Addition to total population results in a difference of +5* | **Total (12-18 y):**  173/844 (weighted)  **Female (12-18 y):**  134/462 (weighted)  **Male (12-18 y):**  39/382 (weighted) | ***Total (12-18 y):****  *-0.12 (-0.25 to 0.01)^3^*  ***Female (12-18 y):***  *-0.17 (-0.32 to -0.02)^3^*  ***Male (12-18 y):***  *-0.70 (-0.89 to -0.51)^3^* | **Total (12-18 y):**  0.81 (0.64 to 1.02)^1^  **Female (12-18 y):**  0.74 (0.56 to 0.97)^1^  **Male (12-18 y):**  0.28 (0.20 to 0.40)^1^ | **Serious** |
| Wright, 2021 [24] | Short Spence Anxiety Scale | No adjustment | General anxiety symptoms  *Additional data were provided by authors* | **Total (10-12 y), mother-rated:**  4.45 (3.45) 109  **Female (10-12 y), mother-rated:**  4.49 (3.35) 109  **Male (10-12 y), mother-rated:**  4.40 (3.59) 90 | **Total (10-12 y), mother-rated:**  4.43 (3.30) 226  **Female (10-12 y), mother-rated:**  4.54 (3.33) 123  **Male (10-12 y), mother-rated:**  4.31 (3.27) 103 | **Total (10-12 y),  mother-rated:**  0.01 (-0.22 to 0.24)³  **Female (10-12 y):**  -0.01 (-0.27 to 0.25)^3^  **Male (10-12 y):**  0.03 (-0.26 to 0.32)^3^ |  | **Serious** |
| Bignardi, 2020 [25] | Revised Child Anxiety and Depression Scale (RCADS)-short form | No adjustment  Adjustment for responder, SES, lockdown* SES | General anxiety symptoms  *Additional data were provided by authors* | **Total (7-12 y),**  **parent-reported:**  -0.10 (0.87) 54  **Female (7-12 y), parent-reported:**  -0.21 (0.90) 32  **Male (7-12y),**  **parent-reported:**  0.07 (0.81) 22 | **Total (7-12 y),**  **parent-reported:**  -0.30 (0.91) 51  **Female (7-12 y), parent-reported:**  -0.24 (0.86) 31  **Male (7-12y),**  **parent-reported:**  -0.38 (1.00) 20 | **Total (7-12 y),**  **parent-reported:**  0.22 (-0.16 to 0.61)³  **Female (7-12 y),**  **parent-reported:**  0.03 (-0.46 to 0.52)³  **Male (7-12y),**  **parent-reported:**  0.49 (-0.13 to 1.11)³  **Total (7-12y):**  -0.06 (-0.34 to 0.22)² |  | **Serious** |

* Effect estimates in italics have been converted using appropriate conversion factors.

1 Effect estimates were self-calculated based on the reported data.

² Effect estimates were so reported in the publication.

³ Additional data for calculation requested.

DP, during pandemic; ED, Emergency departments; M, mean; n, events; N, total; NI, no information; PP, pre-pandemic; SD, Standard deviation; y, years of age

# Table S9. Moderator analysis for total sample with categorical moderators

| **Categorical moderators** | **k** | **Estimate (95% CI)** | **Test of moderators** | **Test for residual heterogeneity** |
| --- | --- | --- | --- | --- |
| Risk of Bias | 16 | 0.32 (0.05 to 0.59) | **p=0.02** | p<0.0001 |
| Symptom reporter | 16 | -0.22 (-0.93 to 0.50) | p=0.55 | p<0.0001 |
| Country | 16 | 0.16 (-0.12 to 0.44) | p=0.17 | p<0.0001 |
| Stringency Index | 16 | 0.10 (-0.28 to 0.48) | p=0.61 | p<0.0001 |
| School Closure Index | 16 | -0.09 (-0.45 to 0.27) | p=0.62 | p<0.0001 |
| Study design | 16 | 0.65 (0.06 to 1.23) | **p=0.03** | p<0.0001 |

# Table S10. Moderator analysis for total sample with continuous moderators

| **Continuous moderators** | **k** | **Estimate (95% CI)** | **Test of moderators** | **Test for residual heterogeneity** |
| --- | --- | --- | --- | --- |
| Age | 16 | 0.09 (0.01 to 0.08) | **p=0.03** | p<0.0001 |
| Gender (% females) | 16 | -0.01 (-0.04 to 0.02) | p=0.36 | p<0.0001 |
| Time of measurement during pandemic | 16 | 0.01 (0.57 to -0.03) | p=0.57 | p<0.0001 |
| Publication year | 16 | 0.11 (0.39 to -0.14) | p=0.38 | p<0.0001 |
| Stringency Index | 16 | 0.00 (-0.01 to 0.02) | p=0.52 | p<0.0001 |
| School Closure Index | 16 | -0.02 (-0.22 to 0.18) | p=0.83 | p<0.0001 |
| Sample size | 16 | 0.00 (-0.00 to 0.00) | p=0.27 | p<0.0001 |

# Table S11. Moderator analysis for female subsample with categorical moderators

| **Categorical moderators** | **k** | **Estimate (95% CI)** | **Test of moderators** | **Test for residual heterogeneity** |
| --- | --- | --- | --- | --- |
| Risk of Bias | 14 | 0.40 (0.17 to 0.62) | **p=0.006** | p<0.0001 |
| Symptom reporter | 14 | -0.03 (-0.77 to 0.71) | p=0.94 | p<0.0001 |
| Country | 14 | -0.04 (-0.08 to 0.01) | p=0.15 | p<0.0001 |
| Stringency Index | 14 | -0.03 (-0.38 to 0.31) | p=0.84 | p<0.0001 |
| School Closure Index | 14 | 0.08 (-0.26 to 0.42) | p=0.64 | p<0.0001 |
| Study design | 14 | 0.64 (0.22 to 1.06) | **p=0.003** | p<0.0001 |

# Table S12. Moderator analysis for female subsample with continuous moderators

| **Continuous moderators** | **k** | **Estimate (95% CI)** | **Test of moderators** | **Test for residual heterogeneity** |
| --- | --- | --- | --- | --- |
| Time of measurement during pandemic | 14 | 0.01 (-0.02 to 0.04) | p=0.51 | p<0.0001 |
| Publication year | 14 | 0.00 (0.98 to -0.23) | p=0.98 | p<0.0001 |
| Stringency Index | 14 | 0.00 (-0.01 to 0.02) | p=0.66 | p<0.0001 |
| School Closure Index | 14 | 0.02 (-0.16 to 0.19) | p=0.86 | p<0.0001 |
| Sample size | 14 | 0.00 (-0.00 to 0.00) | p=0.14 | p<0.0001 |

# Table S13. Moderator analysis for male subsample with categorical moderators

| **Categorical moderators** | **k** | **Estimate (95% CI)** | **Test of moderators** | **Test for residual heterogeneity** |
| --- | --- | --- | --- | --- |
| Risk of Bias | 14 | 0.52 (0.21 to 0.84) | **p=0.0012** | p<0.0001 |
| Symptom reporter | 14 | 0.49 (-0.54 to 1.53) | p=0.35 | p<0.0001 |
| Country | 14 | -0.05 (-0.12 to 0.01) | p=0.09 | p<0.0001 |
| Stringency Index | 14 | -0.02 (-0.53 to 0.50) | p=0.95 | p<0.0001 |
| School Closure Index | 14 | 0.17 (-0.34 to 0.69) | p=0.51 | p<0.0001 |
| Study design | 14 | 0.91 (0.23 to 1.59) | **p=0.01** | p<0.0001 |

# Table S14. Moderator analysis for male subsample with continuous moderators

| **Continuous moderators** | **k** | **Estimate (95% CI)** | **Test of moderators** | **Test for residual heterogeneity** |
| --- | --- | --- | --- | --- |
| Age | 14 | -0.01 (-0.13 to 0.11) | p=0.86 | p<0.0001 |
| Time of measurement during pandemic | 14 | 0.01 (-0.04 to 0.06) | p=0.79 | p<0.0001 |
| Publication year | 14 | -0.13 (-0.47 to 0.21) | p=0.44 | p<0.0001 |
| Stringency Index | 14 | 0.01 (-0.01 to 0.03) | p=0.51 | p<0.0001 |
| School Closure Index | 14 | 0.07 (-0.19 to 0.33) | p=0.60 | p<0.0001 |
| Sample size | 14 | 0.00 (-0.00 to 0.00) | p=0.57 | p<0.0001 |

# Table S15. Sensitivity analysis

| **Population** | **Comparison (number of studies per comparison group in parentheses)** | **Effect estimates  for all studies**  **SMD (95% CI); I²** | **Test for subgroup differences**  **Chi² (p-value)** |
| --- | --- | --- | --- |
| Total | Cohort studies (11) vs  Cross sectional studies (1) | 0.10 (-0.05 to 0.25); 96%  0.75 (0.66 to 0.84); ***only 1 study*** | 52.89 (p<0.001) |
| Total | Adjusted studies (11) vs  unadjusted studies (11) | 0.16 (-0.01 to 0.32), 97%  -0.06 (-0.34 to 0.22); ***only 1 study*** | 1.68 (p=0.19) |
| Total | Unconverted studies (11) vs  converted studies (1) | 0.16 (-0.01 to 0.33), 97%  -0.12 (-0.25 to 0.01); ***only 1 study*** | 6.81 (p=0.009) |

# Table S16. Eggers’ test

| **Population** | **p** |
| --- | --- |
| Total | 0.23 |
| Female | 0.16 |
| Male | 0.32 |

# Figure S1: PRISMA Flow Chart


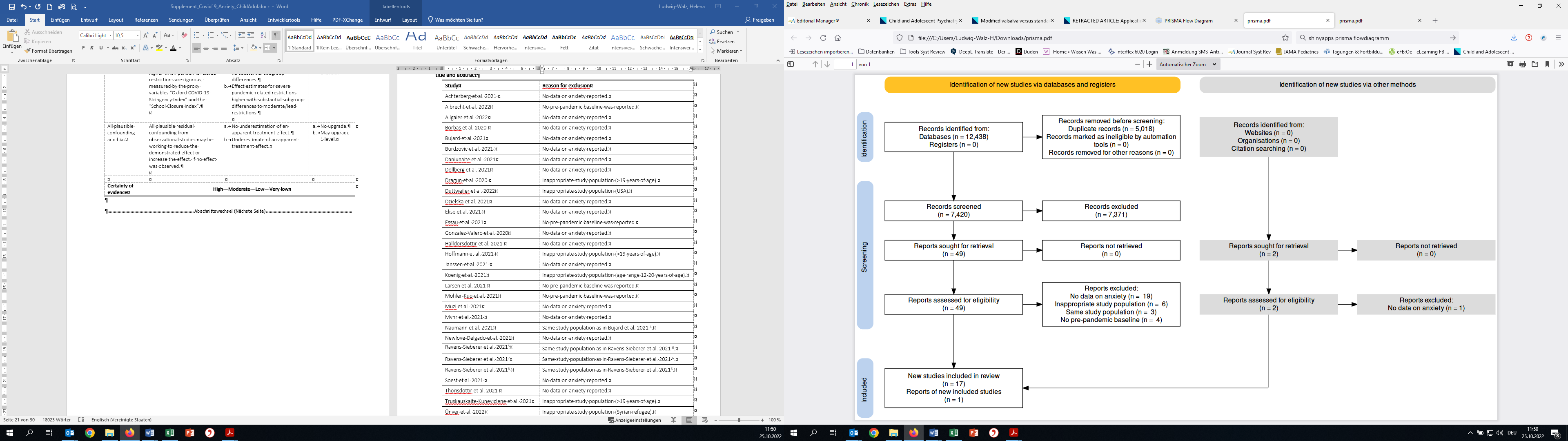


# Figure S2: Traffic-light plots of the domain-level judgements for each individual result


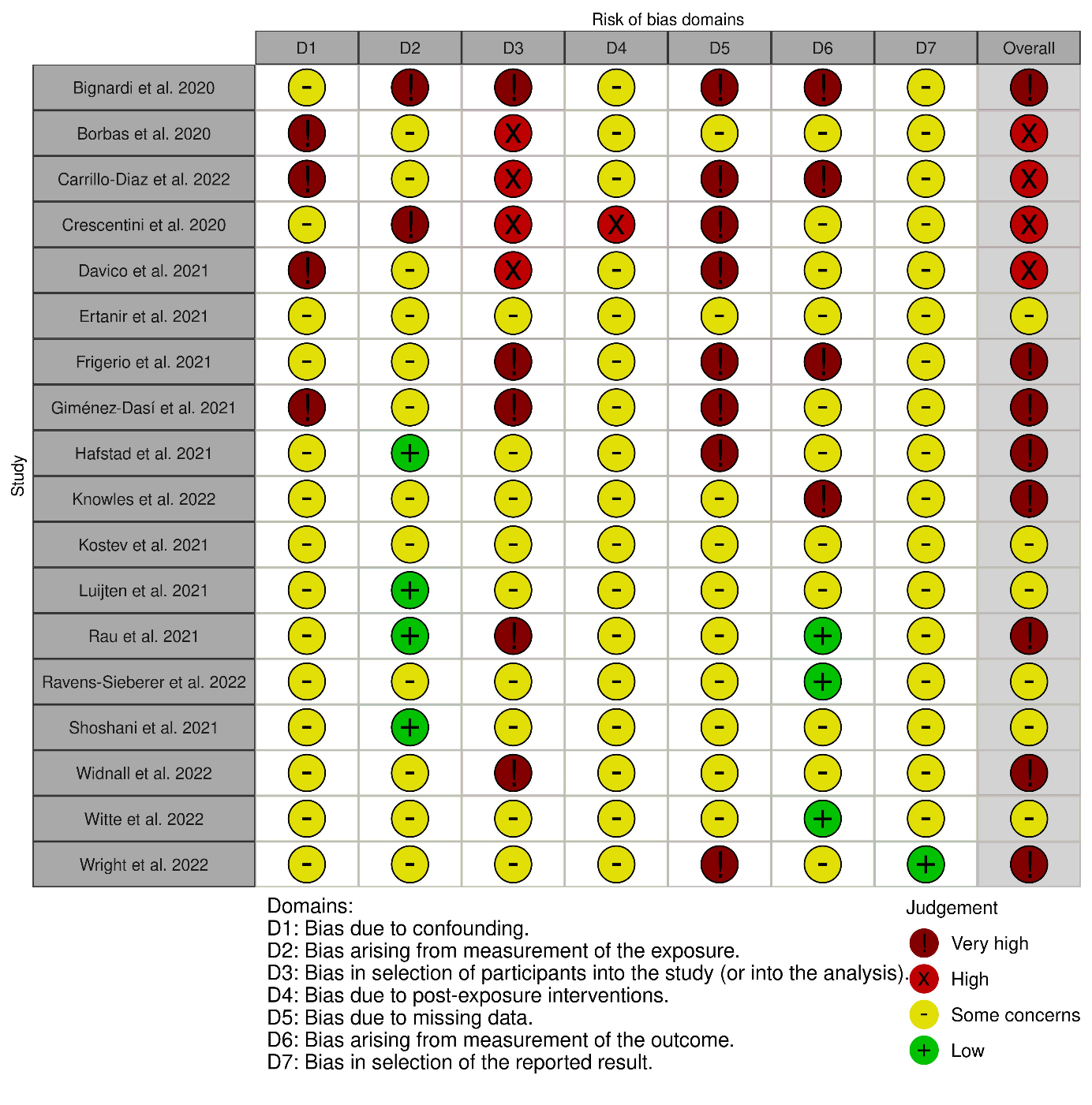


Figure was created using the tool *robvis* [26].

# Figure S3: Weighted-bar plots of the distribution of risk of bias judgements within each bias domain


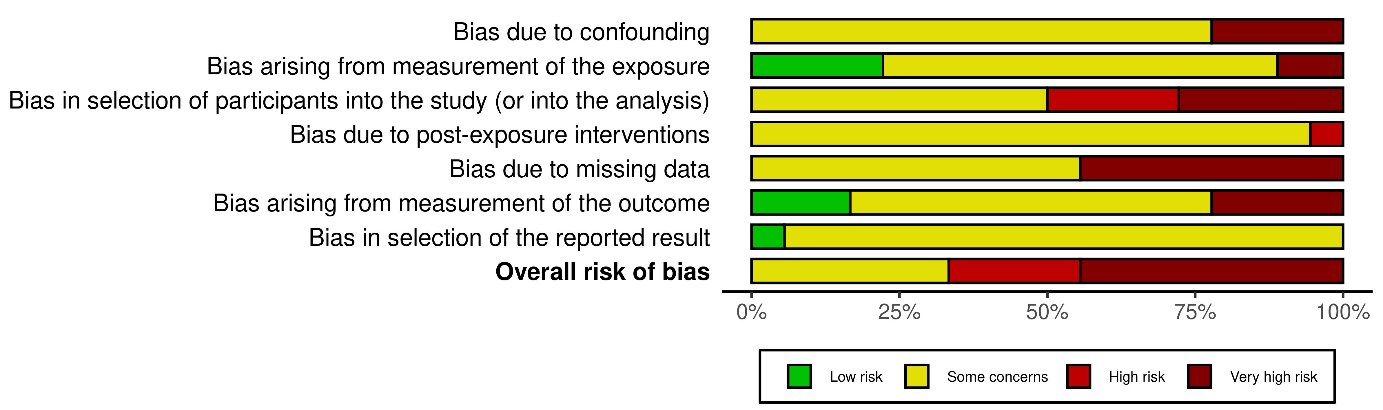


Figure was created using the tool *robvis* [26].

# Figure S4: Forest Plot of Changes in Female General Anxiety Symptoms Comparing Before and During COVID-19 Pandemic


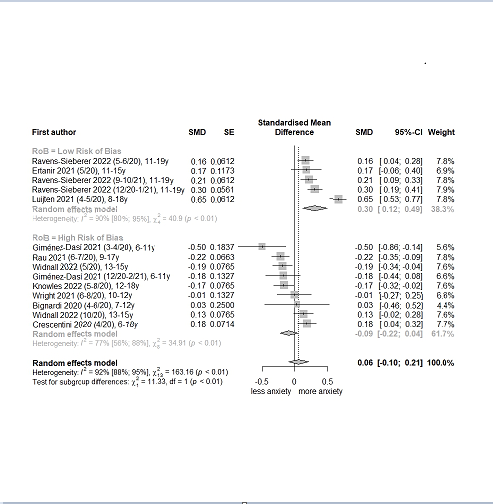


# Figure S5: Forest Plot of Changes in Male General Anxiety Symptoms Comparing Before and During COVID-19 Pandemic


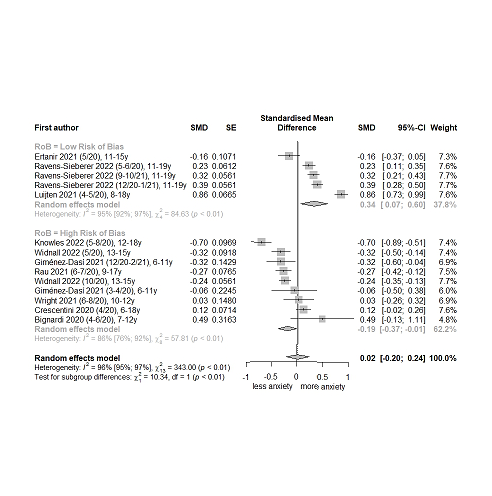


# Figure S6. Forest Plot of Changes in Total (11-15 years) General Anxiety Symptoms Comparing Before and During COVID-19 Pandemic


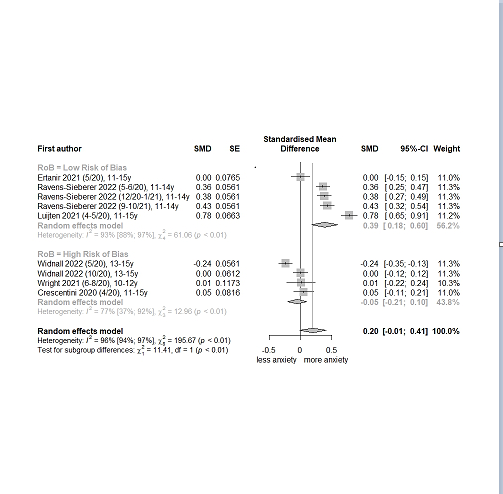


# Figure S7. Forest Plot of Changes in Female (11-15 years) General Anxiety Symptoms Comparing Before and During COVID-19 Pandemic


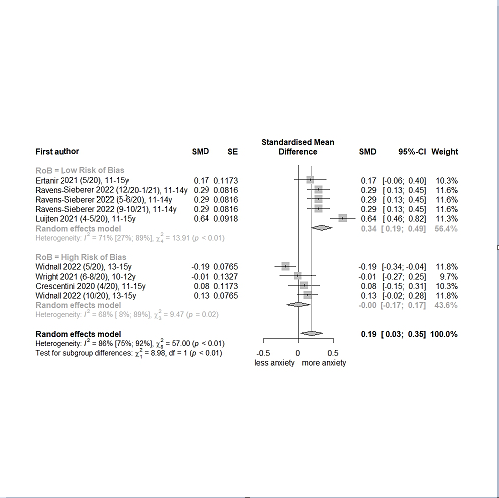


# Figure S8. Forest Plot of Changes in Male (11-15 years) General Anxiety Symptoms Comparing Before and During COVID-19 Pandemic


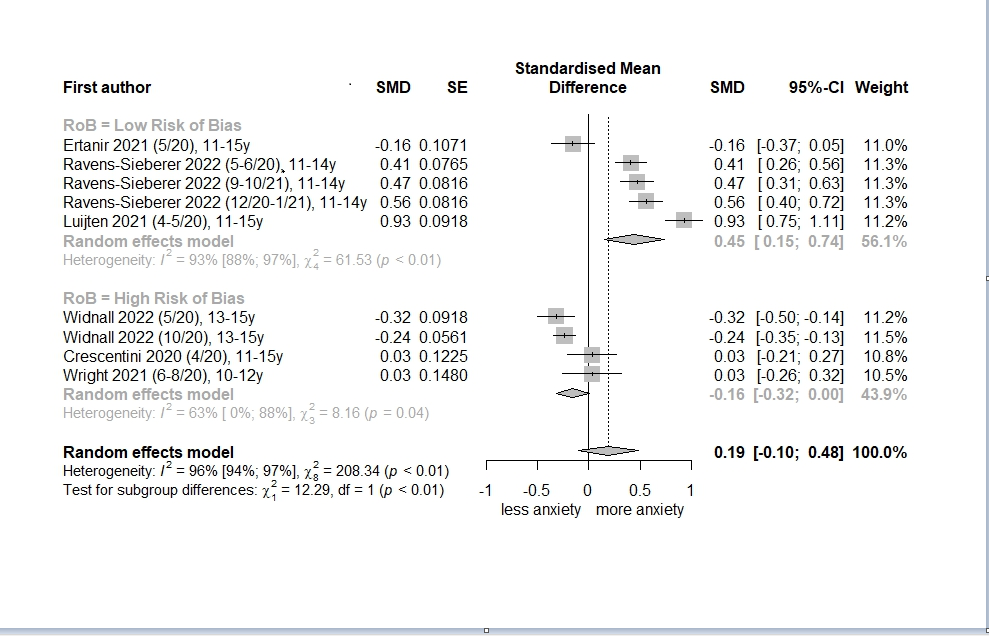


# Figure S9. Forest Plot of Changes in Total (16-19 years) General Anxiety Symptoms Comparing Before and During COVID-19 Pandemic


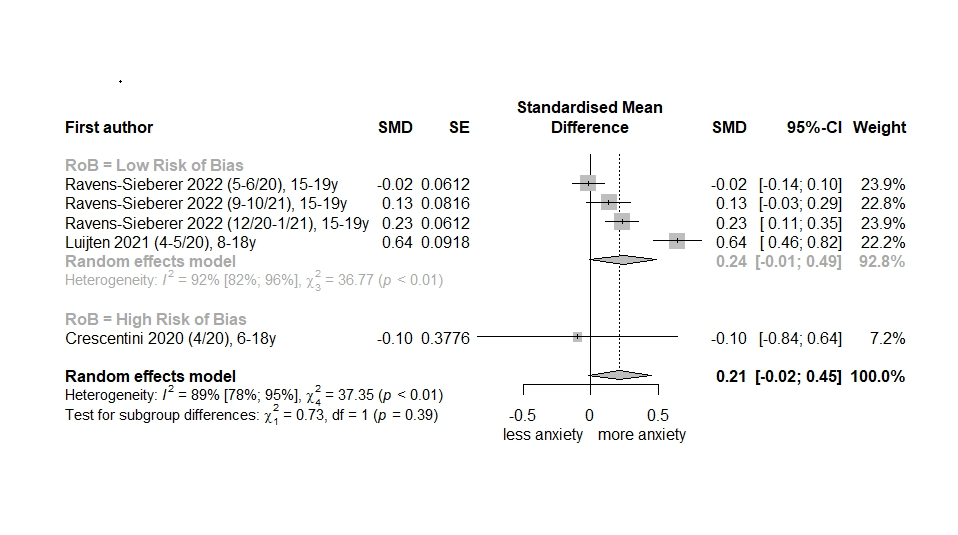


# Figure S10. Forest Plot of Changes in Female (16-19 years) General Anxiety Symptoms Comparing Before and During COVID-19 Pandemic


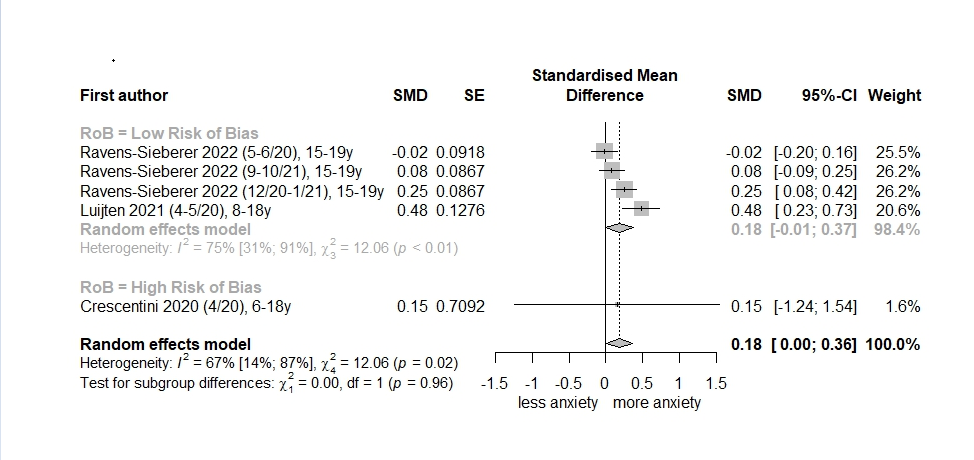


# Figure S11. Forest Plot of Changes in Male (16-19 years) General Anxiety Symptoms Comparing Before and During COVID-19 Pandemic


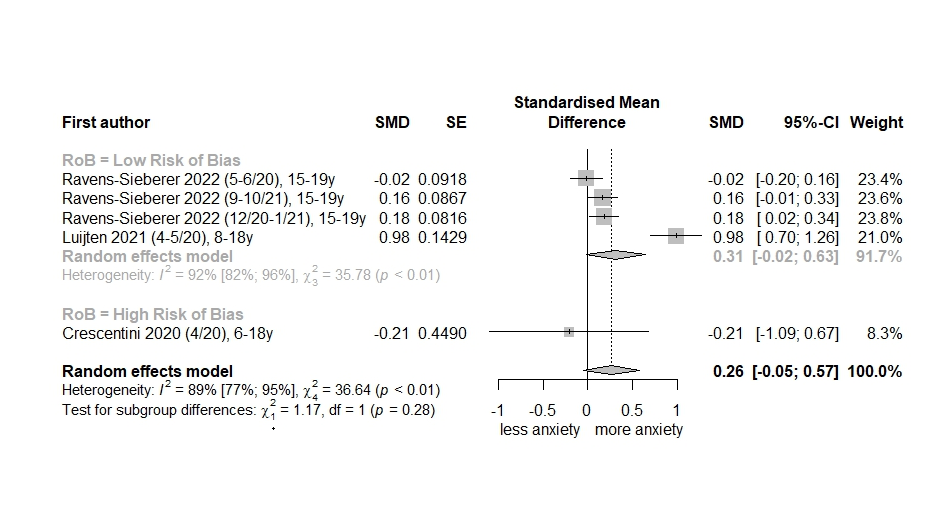


# Figure S12. Forest Plot of Changes in Female Clinically Relevant Anxiety Rates Comparing Before and During COVID-19 Pandemic


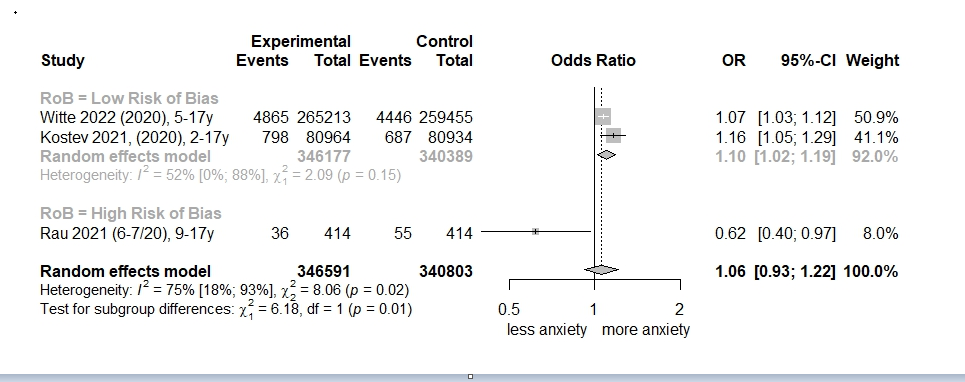


# Figure S13. Forest Plot of Changes in Male Clinically Relevant Anxiety Rates Comparing Before and During COVID-19 Pandemic


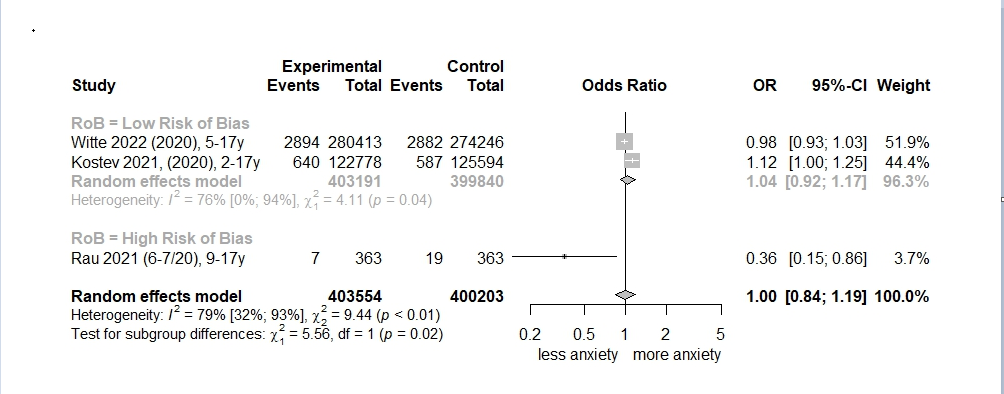


# Figure S14. Funnel Plot of Changes in Total General Anxiety Symptoms Comparing Before and During COVID-19 Pandemic


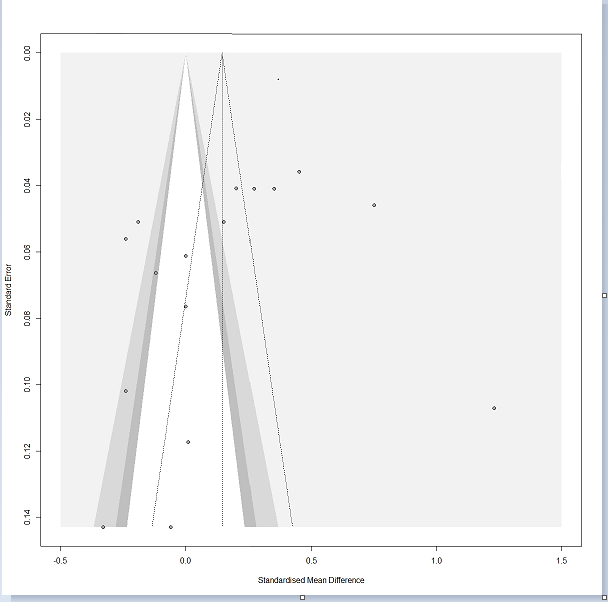


# Figure S15. Funnel Plot of Changes in Female General Anxiety Symptoms Comparing Before and During COVID-19 Pandemic


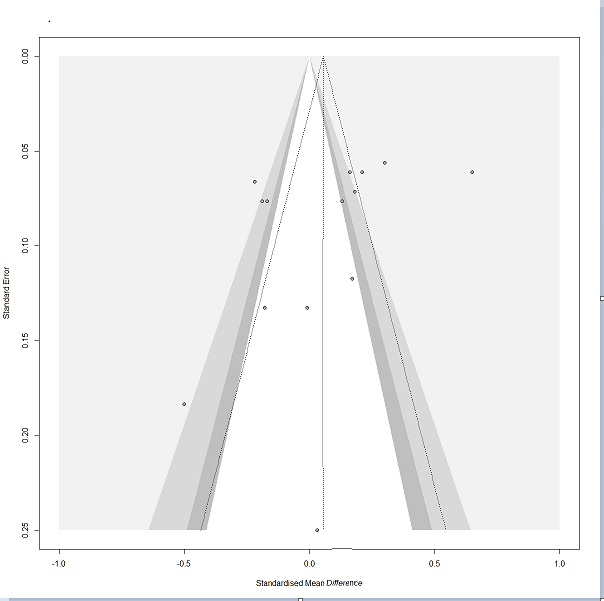


# Figure S16. Funnel Plot of Changes in Male General Anxiety Symptoms Comparing Before and During COVID-19 Pandemic


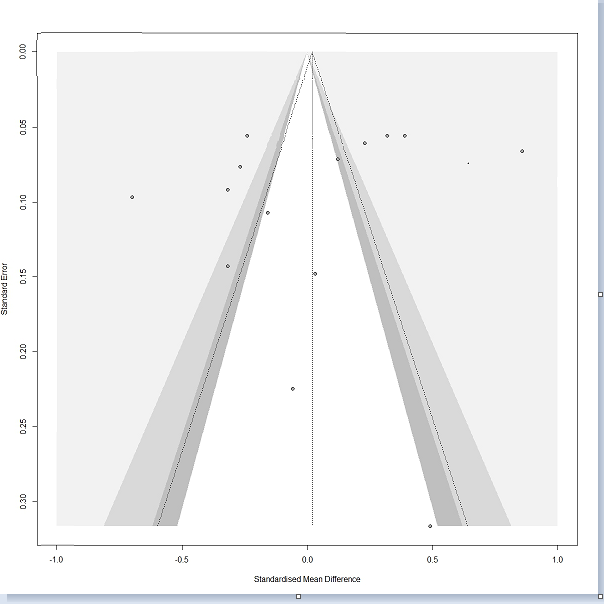


# Figure S17. Funnel Plot of Changes in Total Clinically Relevant Anxiety Symptoms Comparing Before and During COVID-19 Pandemic


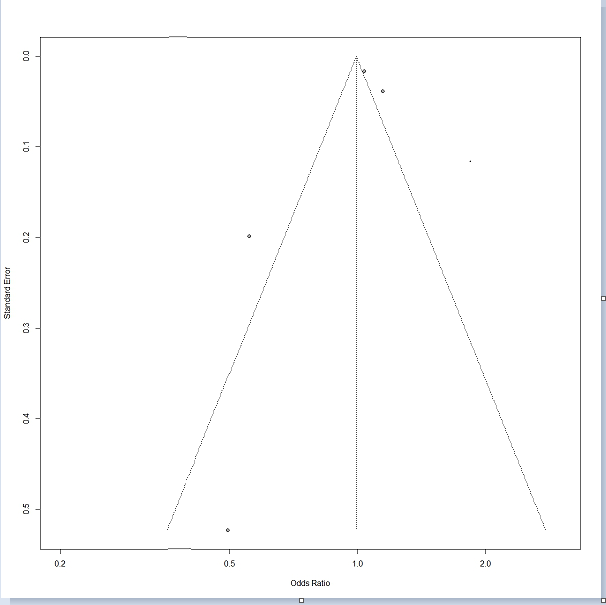


# Figure S18. Funnel Plot of Changes in Female Clinically Relevant Anxiety Symptoms Comparing Before and During COVID-19 Pandemic


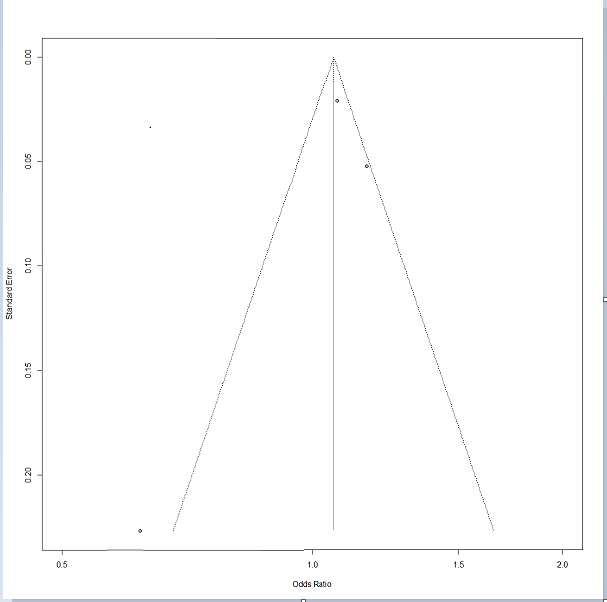


# Figure S19. Funnel Plot of Changes in Male Clinically Relevant Anxiety Symptoms Comparing Before and During COVID-19 Pandemic


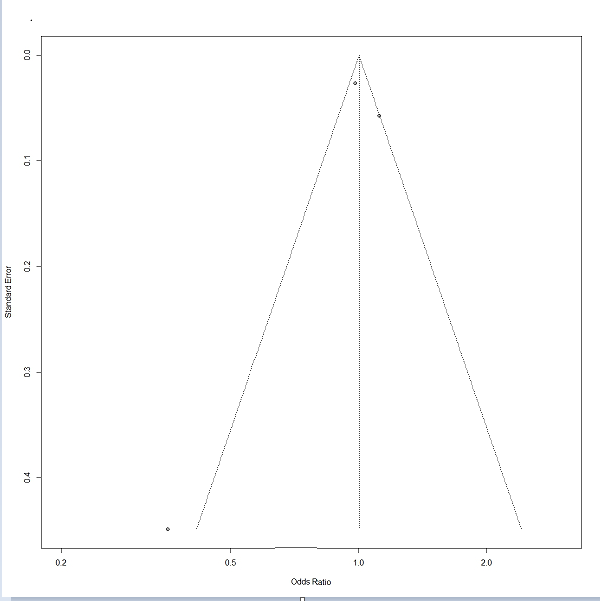


References

1. Morgan RL, Thayer KA, Santesso N, Holloway AC, Blain R, Eftim SE, et al. A risk of bias instrument for non-randomized studies of exposures: A users' guide to its application in the context of GRADE. Environ Int. 2019;122:168–84. doi:10.1016/j.envint.2018.11.004.

2. Schünemann H, Brożek J, Guyatt G, Oxman A. GRADE Handbook: Handbook for grading the quality of evidence and the strength of recommendations using the GRADE approach. Updated October 2013. https://gdt.gradepro.org/app/handbook/handbook.html#h.w6r7mtvq3mjz. Accessed 31 Aug 2022.

3. Schünemann HJ, Cuello C, Akl EA, Mustafa RA, Meerpohl JJ, Thayer K, et al. GRADE guidelines: 18. How ROBINS-I and other tools to assess risk of bias in nonrandomized studies should be used to rate the certainty of a body of evidence. J Clin Epidemiol. 2019;111:105–14. doi:10.1016/j.jclinepi.2018.01.012.

4. Bujard M, den Driesch E von, Kerstin R, Laß I, Thönnissen C, Schumann A, Schneider N. Belastungen von Kindern, Jugendlichen und Eltern in der Corona-Pandemie: BiB.Bevölkerungs.Studien 2/2021. https://www.bib.bund.de/Publikation/2021/pdf/Belastungen-von-Kindern-Jugendlichen-und-Eltern-in-der-Corona-Pandemie.pdf?__blob=publicationFile&v=6. Accessed 29 Aug 2022.

5. Ravens-Sieberer U, Kaman A, Otto C, Adedeji A, Napp A-K, Becker M, et al. Mental health and psychological burden of children and adolescents during the first wave of the COVID-19 pandemic-results of the COPSY study. [Mental health and psychological burden of children and adolescents during the first wave of the COVID-19 pandemic-results of the COPSY study]. Bundesgesundheitsblatt Gesundheitsforschung Gesundheitsschutz. 2021;64:1512–21. doi:10.1007/s00103-021-03291-3.

6. Ravens-Sieberer U, Erhart M, Devine J, Gilbert M, Reiss F, Barkmann C, et al. Child and Adolescent Mental Health During the COVID-19 Pandemic: Results of the Three-Wave Longitudinal COPSY Study. SSRN Journal 2022. doi:10.2139/ssrn.4024489.

7. Ravens-Sieberer U, Kaman A, Erhart M, Devine J, Schlack R, Otto C. Impact of the COVID-19 pandemic on quality of life and mental health in children and adolescents in Germany. Eur Child Adolesc Psychiatry. 2022;31:879–89. doi:10.1007/s00787-021-01726-5.

8. Ravens-Sieberer U, Kaman A, Erhart M, Otto C, Devine J, Löffler C, et al. Quality of life and mental health in children and adolescents during the first year of the COVID-19 pandemic: results of a two-wave nationwide population-based study. Eur Child Adolesc Psychiatry 2021. doi:10.1007/s00787-021-01889-1.

9. Witte J, Zeitler A, Hasemann L. Krankenhausversorgung von Kindern und Jugendlichen während der Pandemie: Fokus: Psychische Erkrankungen. Bielefeld; 2022.

10. Kostev K, Weber K, Riedel-Heller S, Vultée C von, Bohlken J. Increase in depression and anxiety disorder diagnoses during the COVID-19 pandemic in children and adolescents followed in pediatric practices in Germany. Eur Child Adolesc Psychiatry 2021. doi:10.1007/s00787-021-01924-1.

11. Rau L-M, Grothus S, Sommer A, Grochowska K, Claus BB, Zernikow B, Wager J. Chronic Pain in Schoolchildren and its Association With Psychological Wellbeing Before and During the COVID-19 Pandemic. J Adolesc Health. 2021;69:721–8. doi:10.1016/j.jadohealth.2021.07.027.

12. Shoshani A, Kor A. The mental health effects of the COVID-19 pandemic on children and adolescents: Risk and protective factors. Psychol Trauma 2021. doi:10.1037/tra0001188.

13. Frigerio A, Nettuno F, Nazzari S. Maternal mood moderates the trajectory of emotional and behavioural problems from pre- to during the COVID-19 lockdown in preschool children. Eur Child Adolesc Psychiatry 2022. doi:10.1007/s00787-021-01925-0.

14. Davico C, Marcotulli D, Lux C, Calderoni D, Cammisa L, Bondone C, et al. Impact of the COVID-19 Pandemic on Child and Adolescent Psychiatric Emergencies. J Clin Psychiatry 2021. doi:10.4088/JCP.20m13467.

15. Crescentini C, Feruglio S, Matiz A, Paschetto A, Vidal E, Cogo P, Fabbro F. Stuck Outside and Inside: An Exploratory Study on the Effects of the COVID-19 Outbreak on Italian Parents and Children's Internalizing Symptoms. Front Psychol. 2020;11:586074. doi:10.3389/fpsyg.2020.586074.

16. Luijten MAJ, van Muilekom MM, Teela L, Polderman TJC, Terwee CB, Zijlmans J, et al. The impact of lockdown during the COVID-19 pandemic on mental and social health of children and adolescents. Qual Life Res. 2021;30:2795–804. doi:10.1007/s11136-021-02861-x.

17. Hafstad GS, Sætren SS, Wentzel-Larsen T, Augusti E-M. Adolescents' symptoms of anxiety and depression before and during the Covid-19 outbreak - A prospective population-based study of teenagers in Norway. Lancet Reg Health Eur. 2021;5:100093. doi:10.1016/j.lanepe.2021.100093.

18. Giménez-Dasí M, Quintanilla L, Fernández-Sánchez M. Longitudinal Effects of the Pandemic and Confinement on the Anxiety Levels of a Sample of Spanish Children in Primary Education. Int J Environ Res Public Health 2021. doi:10.3390/ijerph182413063.

19. Carrillo-Diaz M, Ortega-Martínez AR, Romero-Maroto M, González-Olmo MJ. Lockdown impact on lifestyle and its association with oral parafunctional habits and bruxism in a Spanish adolescent population. Int J Paediatr Dent. 2022;32:185–93. doi:10.1111/ipd.12843.

20. Ertanir B, Kassis W, Garrote A. Longitudinal Changes in Swiss Adolescent's Mental Health Outcomes from before and during the COVID-19 Pandemic. Int J Environ Res Public Health 2021. doi:10.3390/ijerph182312734.

21. Borbás R, Fehlbaum LV, Dimanova P, Negri A, Arudchelvam J, Schnider CB, Raschle NM. Mental well-being during the first months of Covid-19 in adults and children: behavioral evidence and neural precursors. Sci Rep. 2021;11:17595. doi:10.1038/s41598-021-96852-0.

22. Widnall E, Winstone L, Plackett R, Adams EA, Haworth CMA, Mars B, Kidger J. Impact of School and Peer Connectedness on Adolescent Mental Health and Well-Being Outcomes during the COVID-19 Pandemic: A Longitudinal Panel Survey. Int J Environ Res Public Health 2022. doi:10.3390/ijerph19116768.

23. Knowles G, Gayer-Anderson C, Turner A, Dorn L, Lam J, Davis S, et al. Covid-19, social restrictions, and mental distress among young people: a UK longitudinal, population-based study. J Child Psychol Psychiatry 2022. doi:10.1111/jcpp.13586.

24. Wright N, Hill J, Sharp H, Pickles A. Interplay between long-term vulnerability and new risk: Young adolescent and maternal mental health immediately before and during the COVID-19 pandemic. JCPP Adv. 2021;1:e12008. doi:10.1111/jcv2.12008.

25. Bignardi G, Dalmaijer ES, Anwyl-Irvine AL, Smith TA, Siugzdaite R, Uh S, Astle DE. Longitudinal increases in childhood depression symptoms during the COVID-19 lockdown. Arch Dis Child 2020. doi:10.1136/archdischild-2020-320372.

26. McGuinness LA, Higgins JPT. Risk-of-bias VISualization (robvis): An R package and Shiny web app for visualizing risk-of-bias assessments. Res Synth Methods. 2021;12:55–61. doi:10.1002/jrsm.1411.
